# Supplementary material for: Pan-cancer analysis of tissue and single-cell HIF-pathway activation using a conserved gene signature
Source: Cell Rep. 2022 Nov 15;41(7):111652. doi: 10.1016/j.celrep.2022.111652 (PMC9869179; doi:10.1016/j.celrep.2022.111652)
Supplement: Document S1. Figures S1–S8 [file mmc1.pdf]

Cell Reports, Volume *41*

Supplemental information

Pan-cancer analysis of tissue and single-cell HIF-pathway activation using a conserved gene signature

Olivia Lombardi, Ran Li, Silvia Halim, Hani Choudhry, Peter J. Ratcliffe, and David R. Mole

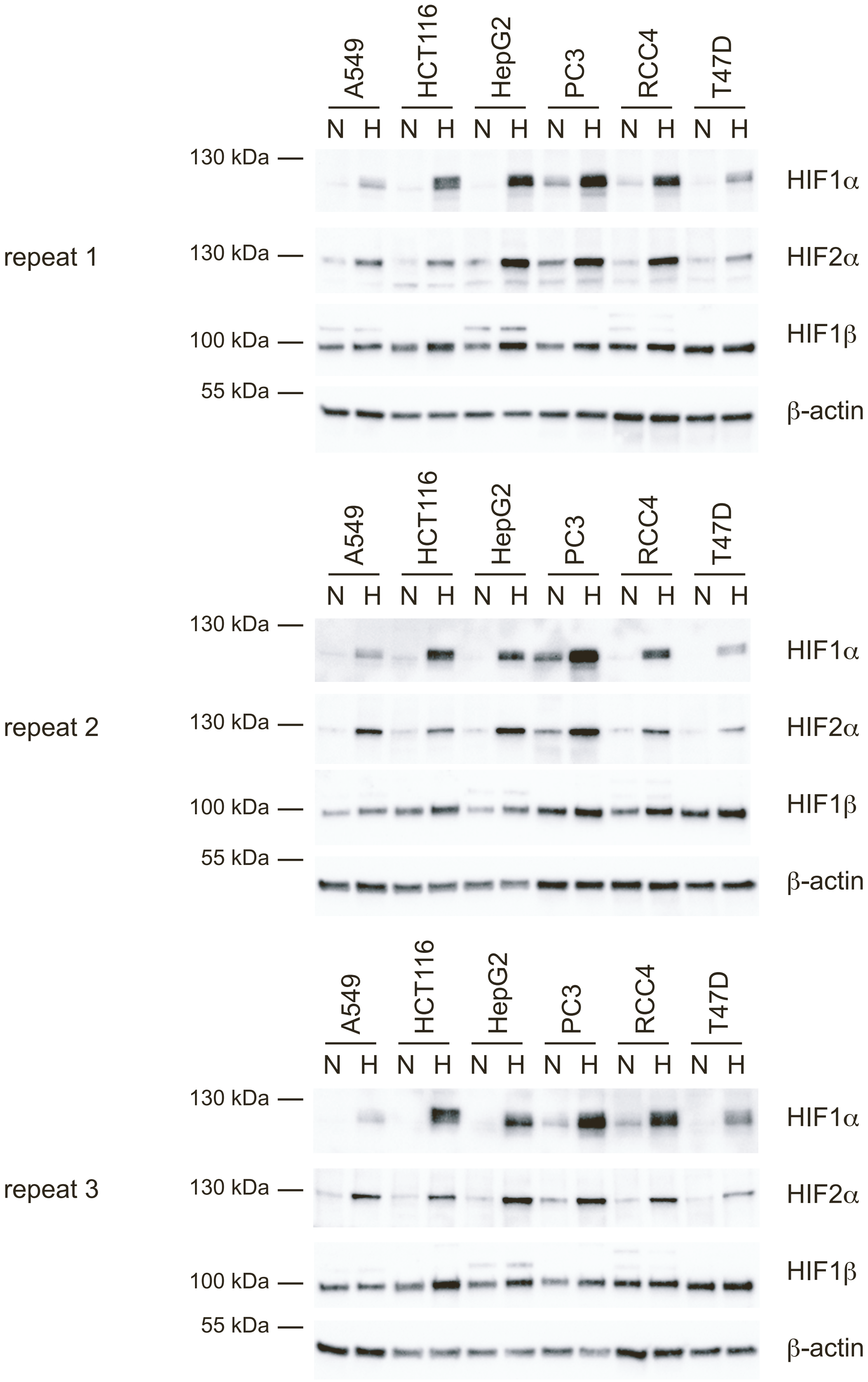

Supplemental Figure 1. HIF subunit levels in cell lines used. Related to Figure 1. Immunoblot showing relative HIF-1α, HIF-2α and HIF-1β protein levels in A549, HCT116, HepG2, PC3, RCC4 and T47D cells.

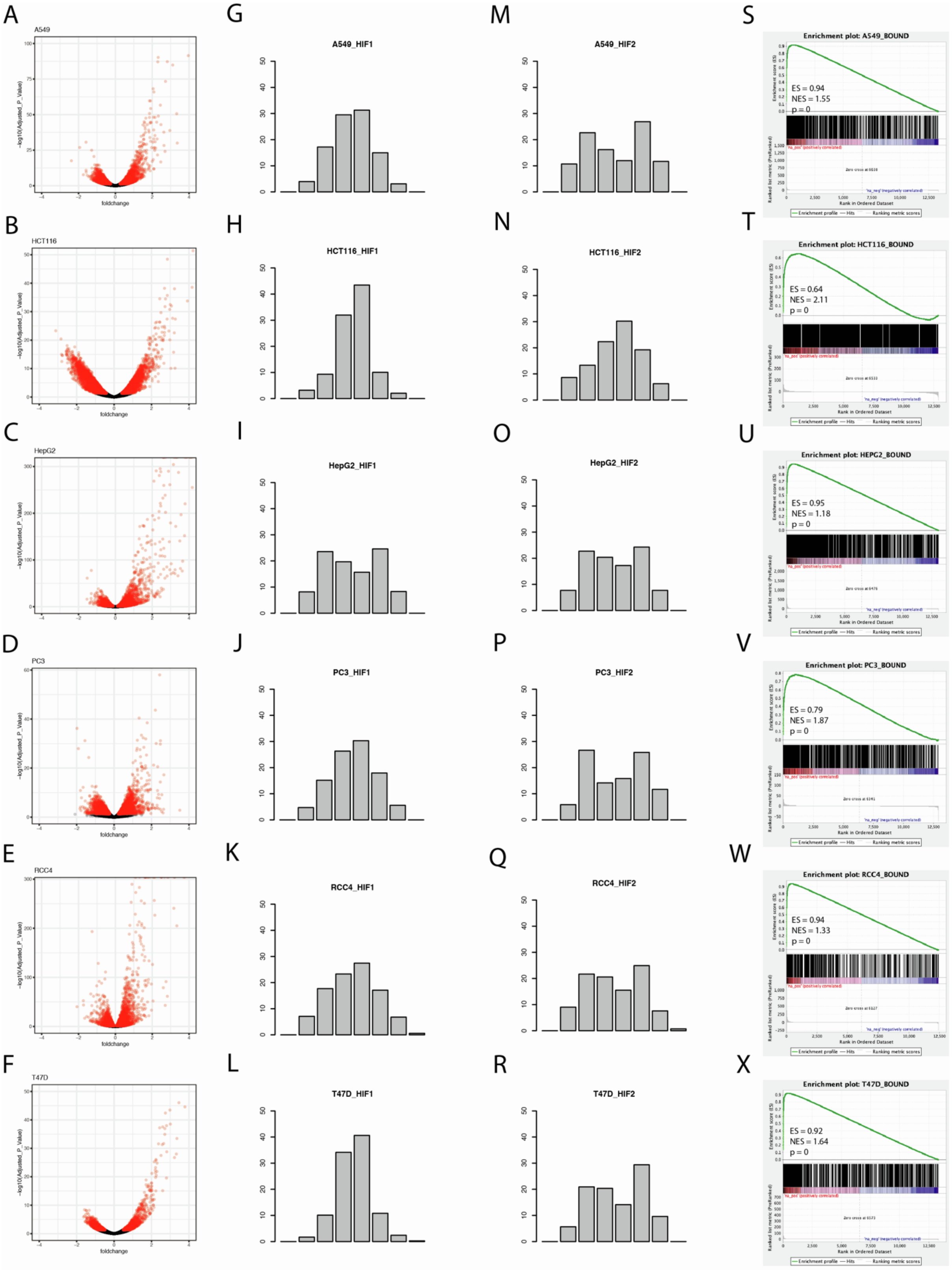

Supplemental Figure 2. Summary of RNA-seq and ChIP-seq analyses in cell lines. Related to Figure 1. Volcano plots showing log2(fold-change) and -log10(p-value) for gene expression in hypoxia and normoxia in RNA-seq analysis of (A) A549, (B) HCT116, (C) HepG2, (D) PC3, (E) RCC4+VHL and (F) T47D cells. Histograms showing frequency distribution of distance to nearest TSS for (G-L) canonical HIF-1 binding sites and (M-R) canonical HIF-2 binding sites. (S-

X) Gene set enrichment analysis (GSEA) showing the enrichment of HIF-bound genes amongst genes induced, but not suppressed by hypoxia.

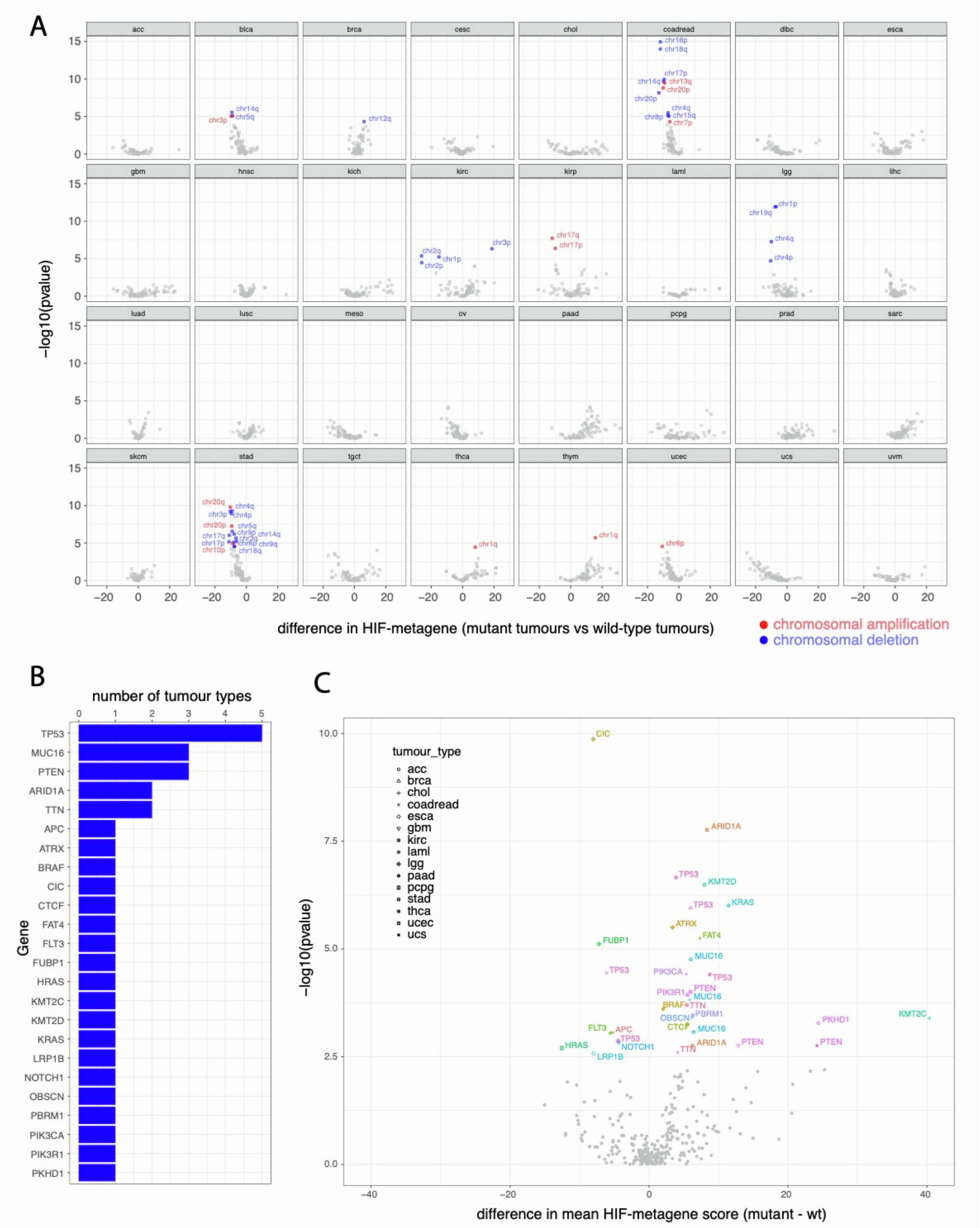

Supplemental Figure 3. Association between genetic mutation and HIF-metagene in tumors from the TCGA database. Related to Figures 3 and 4**.** (A) Volcano plots showing the association between chromosomal arm-level copy number alterations and HIF-metagene score. Red dots show the effect of chromosomal amplification, blue dots show the effect of chromosomal deletion and grey dots show non-significant associations. (B) The number of tumor types in which commonly mutated genes are associated with significantly altered HIF-metagene. (C) Volcano plot showing association between gene mutation and HIF-metagene score for the 10 most commonly mutated genes in each tumor type.

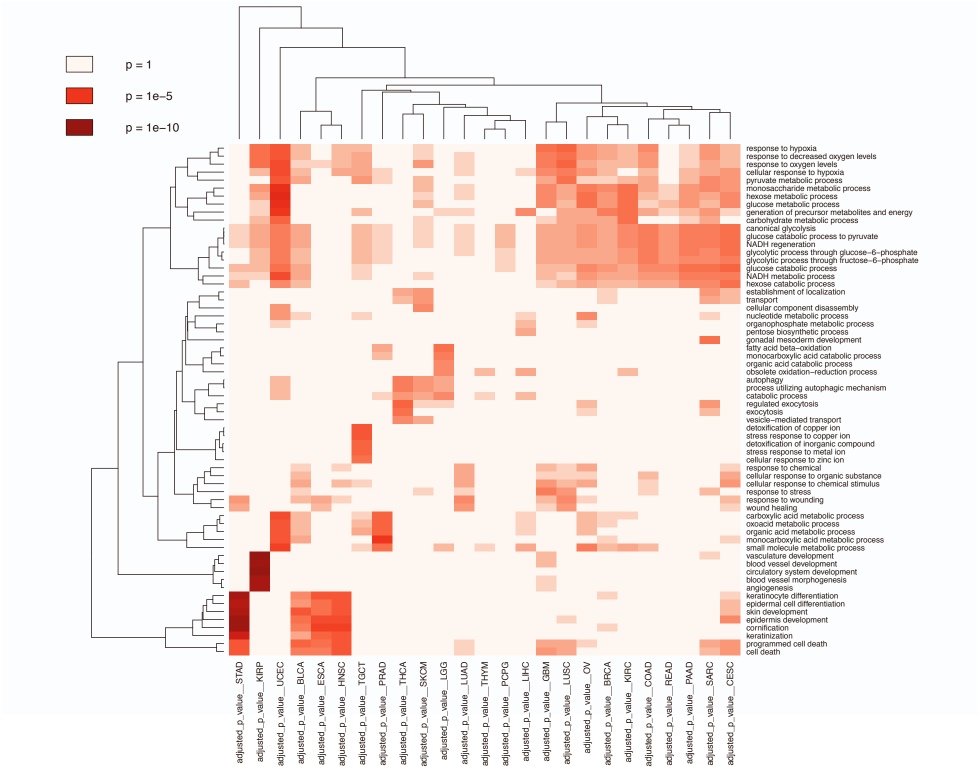

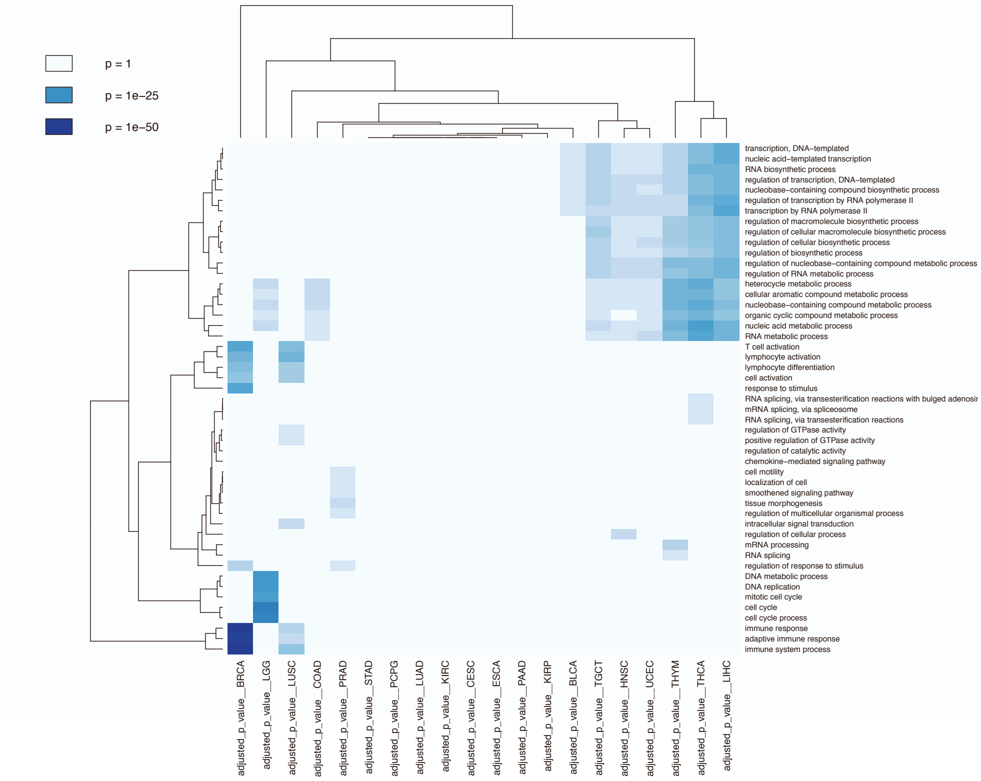

Supplemental Figure 4. Pathway enrichment for genes correlating with HIF-metagene in tumors from the TCGA database. Related to Figures 3 and 4. Heatmap showing -log10(p-value) for pathways enriched amongst (A) genes that positively correlate with the HIF-metagene in each tumor type using RNA-seq analyses from the TCGA for 9,760 tumors drawn from 32 cancer categories and (B) genes that negatively correlate with the HIF-metagene.

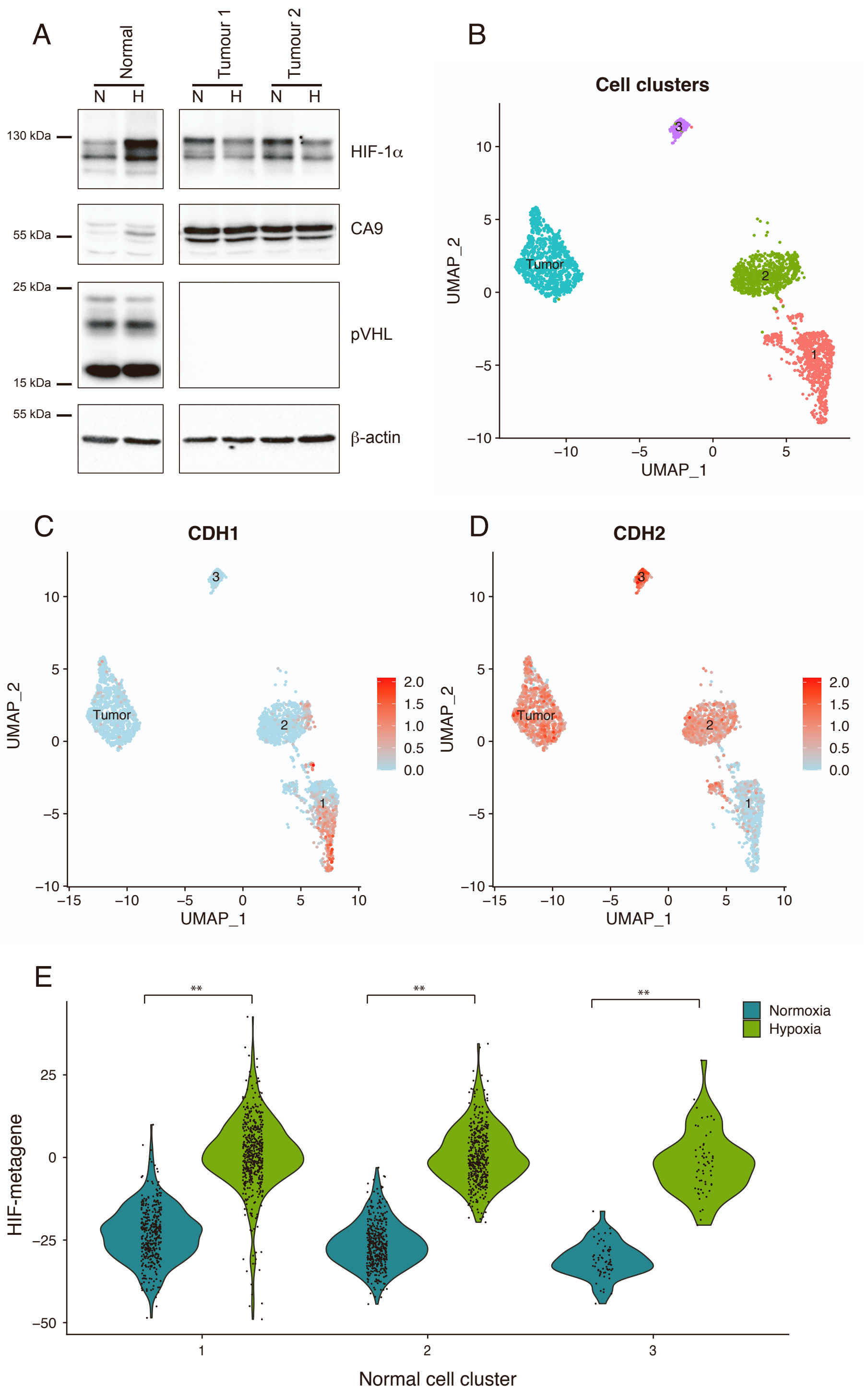

Supplemental Figure 5. Subgroup analysis of individual clusters in scRNA-seq analysis of cultured normal cells. Related to Figure 5. (A) Immunoblot showing HIF-1α, CA9, pVHL and β-actin protein levels in normoxia and hypoxia. UMAP plots showing (A) sample of origin, (B) individual clusters denoted 1=distal tubular epithelial cells, 2=proximal tubular epithelial cells, 3=non-epithelial cells and tumor=ccRCC cancer cells, (C) expression of the distal tubular marker, CDH1, and (D) expression of the proximal tubular marker, CDH2. (E) Violin plot showing HIF-metagene expression in cells from each normal cell cluster incubated in normoxia and 0.5% hypoxia for 16 hours (**, $p<10^{-16}$, Wilcoxon rank sum).

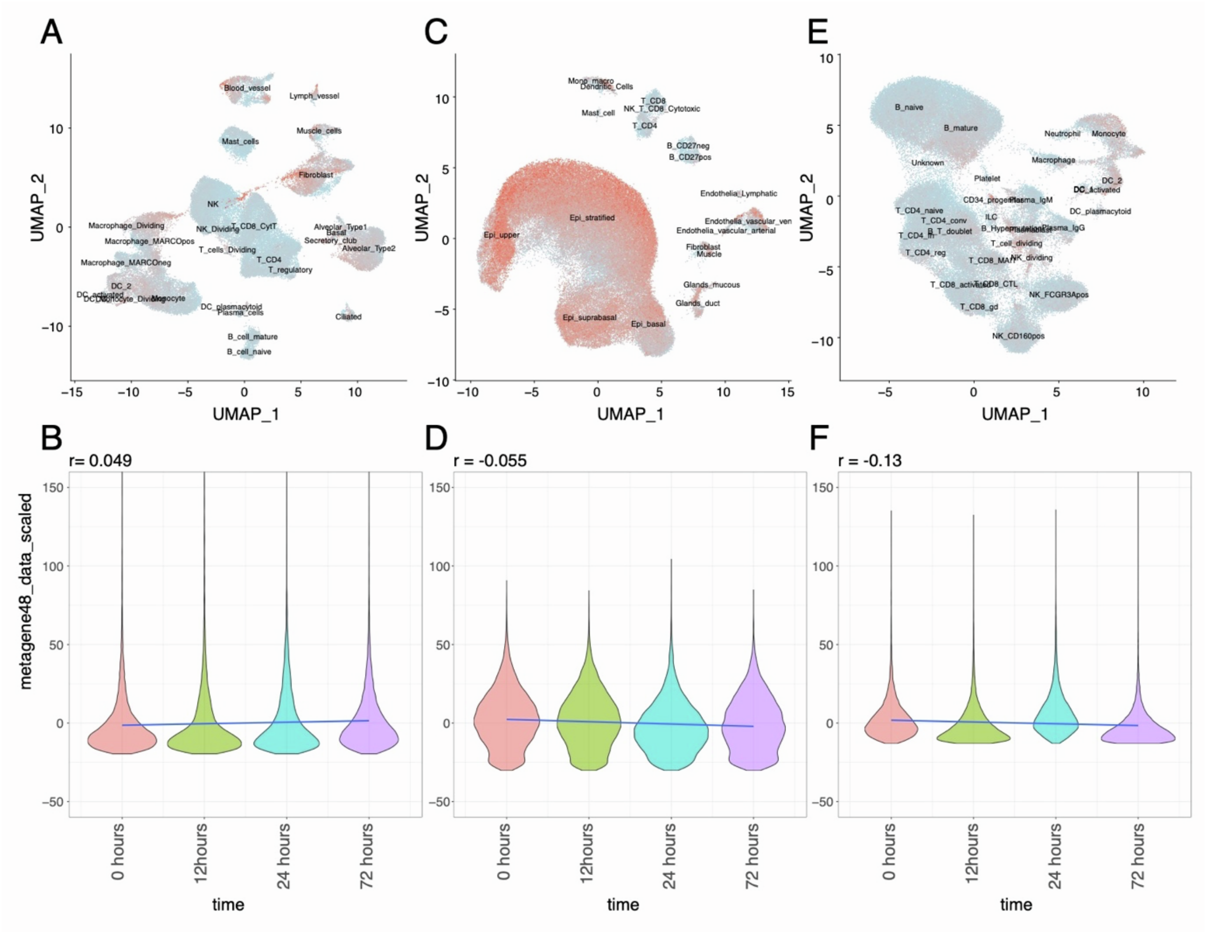

Supplemental Figure 6. **The effect of cold ischemic storage on HIF-metagene in scRNA-seq**. Related to Figures 5 and 6. (A) UMAP plot and (B) violin plot showing HIF-metagene scores in scRNAseq analysis of cells from lung samples subjected to 0, 12, 24 and 72 hours cold ischemic storage prior to processing (Madissoon et al). The same analyses in cells from (C and D) esophagus samples and (E and F) spleen samples.

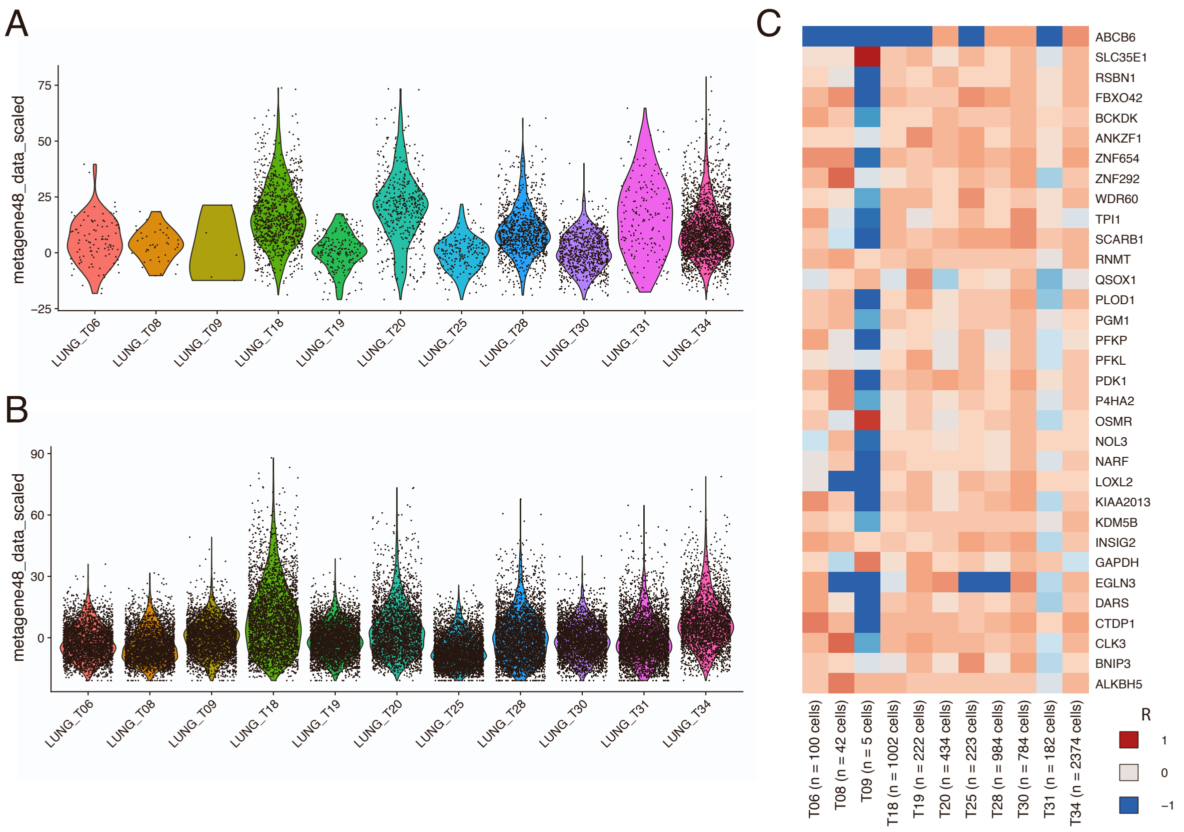

Supplemental Figure 7. HIF-metagene in individual early-stage lung cancer samples. (A) Violin plot showing HIF-metagene expression in tumor cells from each early-stage lung cancer sample in Kim et al. (B) Violin plot showing HIF-metagene expression in non-tumor cells from the same samples. (C) Heatmap showing Pearson correlation coefficient between the HIF-metagene and second-tier HIF-target genes (identified in 5/6 cancer cell lines) in tumor cells from each, individual early-stage lung cancer sample.

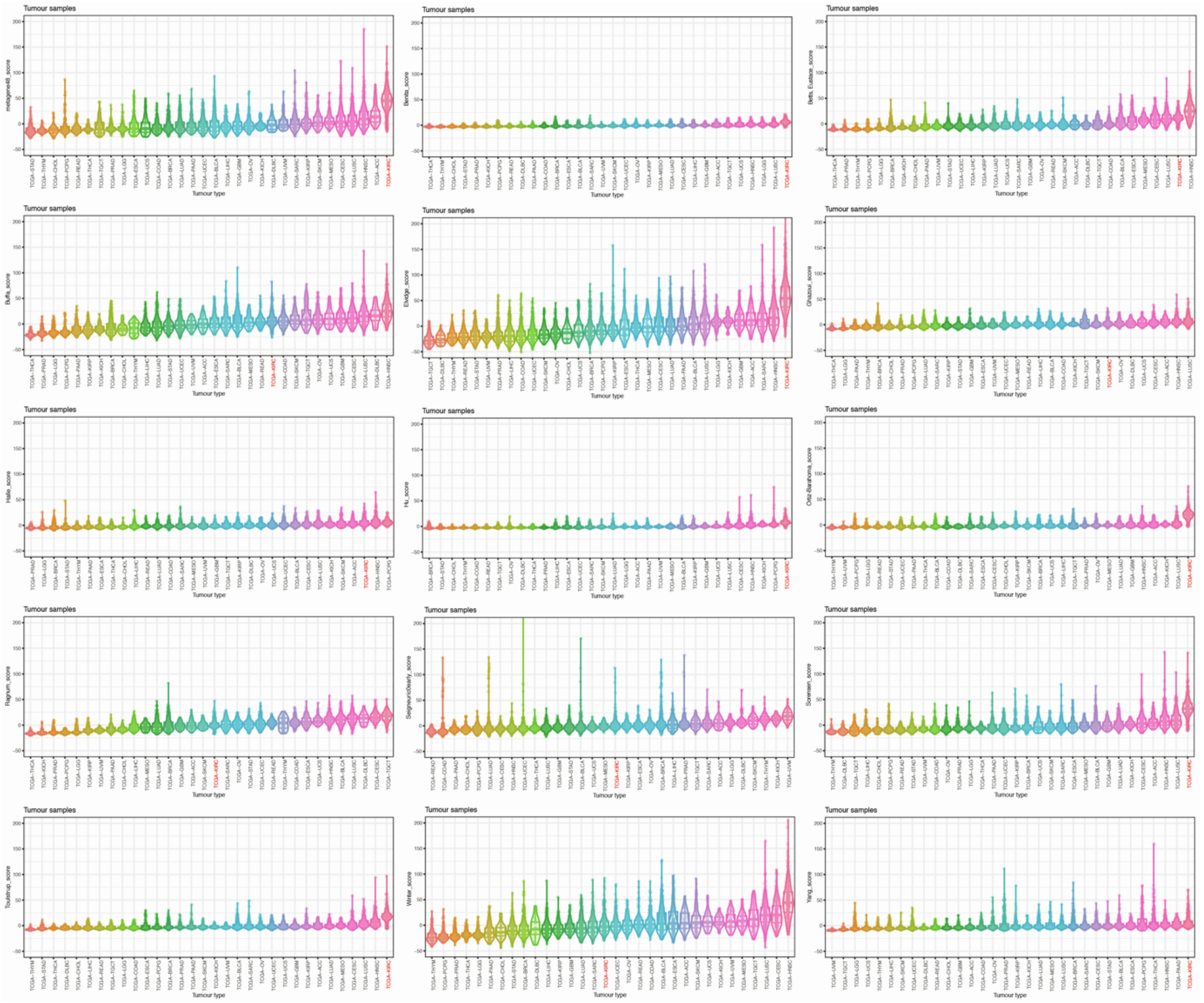

Supplemental Figure 8. Hypoxic gene expression signatures in tumors from the TCGA database. Scatter/box-and-whisker/violin plots showing metagene expression in RNA-seq analysis of 9,760 tumors from the TCGA database using genes from (A) our 48-gene HIF-metagene (B) Benita, (C) Betts and Eustace, (D) Buffa, (E) Elvidge, (F) Ghazoui, (G) Halle, (H) Hu, (I) Ortiz-Barahoma, (J) Ragnum, (K) Seineuric, (L) Sorensen, (M) Toustrup, (N) Winter and (O) Yang. Tumors are grouped according to tumor type and ranked according to median expression for that tumor type.
