# Supplementary material for: Pan-cancer analysis of tissue and single-cell HIF-pathway activation using a conserved gene signature
Source: Cell Rep. 2022 Nov 15;41(7):111652. doi: 10.1016/j.celrep.2022.111652 (PMC9869179; doi:10.1016/j.celrep.2022.111652)
Supplement: Document S2. Article plus supplemental information [file mmc3.pdf]

Cell Reports

Resource

Pan-cancer analysis of tissue and single-cell HIF-pathway activation using a conserved gene signature

Graphical abstract

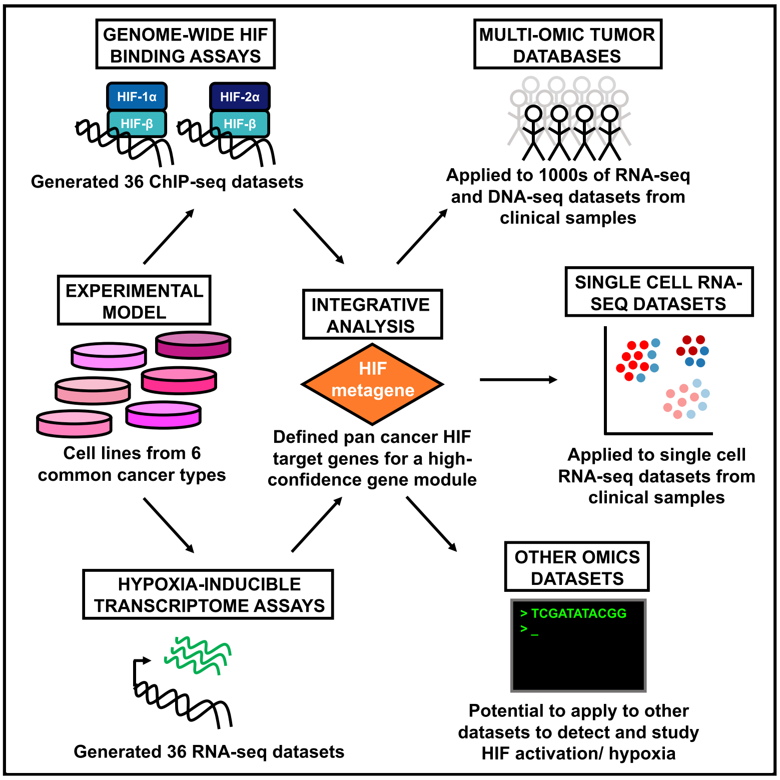

Highlights

- ChIP-seq and RNA-seq datasets are used to identify HIF target genes in multiple cancers
- Consensus HIF target genes are used as a pan-cancer HIF metagene
- The HIF metagene can be used as an endogenous marker of HIF activity/hypoxia
- The HIF metagene allows analysis of tumor samples via bulk and single-cell datasets

Authors

Olivia Lombardi, Ran Li, Silvia Halim, Hani Choudhry, Peter J. Ratcliffe, David R. Mole

Correspondence

david.mole@ndm.ox.ac.uk

In brief

Lombardi et al. define a consensus set of HIF target genes using 72 ChIP-seq and RNA-seq datasets and use this as an “HIF metagene” to detect HIF activation/ hypoxia in clinical samples. This enables investigation of tumor- and cell-type-specific responses to HIF in bulk and single-cell RNA-seq datasets.

Lombardi et al., 2022, Cell Reports *41*, 111652
November 15, 2022 © 2022 The Authors.
https://doi.org/10.1016/j.celrep.2022.111652

CellPress

Cell Reports

CellPress
OPEN ACCESS

Resource

Pan-cancer analysis of tissue and single-cell HIF-pathway activation using a conserved gene signature

Olivia Lombardi,[1] Ran Li,[1] Silvia Halim,[1] Hani Choudhry,[2] Peter J. Ratcliffe,[3,4] and David R. Mole[1,5,*]
[1]NDM Research Building, University of Oxford, Old Road Campus, Headington, Oxford OX3 7FZ, UK
[2]Department of Biochemistry, Faculty of Science, Center of Innovation in Personalized Medicine, King Fahd Center for Medical Research, King Abdulaziz University, Jeddah, Saudi Arabia
[3]Ludwig Institute for Cancer Research, University of Oxford, Old Road Campus, Headington, Oxford OX3 7FZ, UK
[4]The Francis Crick Institute, 1 Midland Road, London NW1 1AT, UK
[5]Lead contact
*Correspondence: david.mole@ndm.ox.ac.uk
https://doi.org/10.1016/j.celrep.2022.111652

SUMMARY

Activation of cellular hypoxia pathways, orchestrated by HIF (hypoxia-inducible factor) transcription factors, is a common feature of multiple tumor types, resulting from microenvironment factors and oncogenic mutation. Although they help drive many of the "hallmarks" of cancer and are associated with poor outcome and resistance to therapy, the transcriptional targets of HIF vary considerably depending on the cell type. By integrating 72 genome-wide assays of HIF binding and transcriptional regulation from multiple cancer types, we define a consensus set of 48 HIF target genes that is highly conserved across cancer types and cell lineages. These genes provide an effective marker of HIF activation in bulk and single-cell transcriptomic analyses across a wide range of cancer types and in malignant and stromal cell types. This allows the tissue-orchestrated responses to the hypoxic tumor microenvironment and to oncogenic HIF activation to be deconvoluted at the tumor and single-cell level.

INTRODUCTION

Low levels of tissue oxygen (hypoxia) arise when oxygen consumption, driven by aerobic metabolism and cell proliferation, exceeds the ability of the vasculature to deliver oxygenated blood to the region. Intra-tumor hypoxia is a common feature of many solid malignant tumors (Vaupel et al., 2004), in which dysregulated cell proliferation often outstrips the ability to develop new functional blood vessels. Hypoxia pathways may also be activated in response to mutation or dysregulation of key tumor suppressor genes or oncogenes (Maxwell et al., 2001; Semenza, 2003). In many types of cancer, tumor hypoxia is associated with a clinically aggressive phenotype and resistance to therapy (Hockel and Vaupel, 2001; Semenza, 2010; Vaupel and Mayer, 2007). Hypoxia helps drive many of the "hallmarks" of cancer, including cell proliferation, apoptosis, metabolism, immune responses, genomic instability, vascularization, and invasion and metastasis (Hanahan and Weinberg, 2011; Semenza, 2012b). The key mediator of cellular responses to hypoxia is the family of HIF (hypoxia-inducible factor) transcription factors. There are three known members of the HIF family, HIF-1, HIF-2, and HIF-3. Each comprises a heterodimer of a regulated alpha subunit, HIF-1α, HIF-2α (also known as EPAS1 - endothelial PAS domain protein 1), or HIF-3α, together with a common constitutive beta subunit, HIF-1β (also known as ARNT - aryl hydrocarbon receptor nuclear translocator) (Kaelin and Ratcliffe, 2008; Semenza, 2012a). HIF-1α and HIF-2α share similar DNA binding, dimerization, regulatory, and transactivation domains with well-established roles in transcriptional regulation, but little is currently known about the function and role of HIF-3 in oxygen homeostasis (Duan, 2016). When oxygen is abundant, a family of prolyl hydroxylases, PHD1–PHD3 (also known as EGLN1–EGLN3) hydroxylate key prolyl residues on HIF-1α and HIF-2α, which facilitates its binding to the von Hippel-Lindau (VHL)-ubiquitin-E3 ligase complex and leads to its rapid degradation (Epstein et al., 2001; Ivan et al., 2001; Jaakkola et al., 2001). Oxygen-dependent hydroxylation of an asparaginyl residue also blocks interaction of HIF with transcriptional co-activators, such as p300 (Hewitson et al., 2002; Lando et al., 2002; Mahon et al., 2001). When oxygen is limited, hydroxylation is restricted, and HIF is stabilized. HIF then binds consensus 5′-(A/G)CGTG-3′ hypoxia response element (HRE) motifs, enhancing transcription of its target genes (Wenger et al., 2005). In clear cell renal cell cancer (ccRCC), these pathways are constitutively active because of loss-of-function mutations of the VHL tumor suppressor gene, which forms part of the E3 ligase central to regulation of HIF (Maxwell et al., 1999).

Genes activated by the HIF transcriptional pathway act to increase oxygen delivery to the cell (e.g., *VEGFA*, *EPO*) and reduce its oxygen consumption (e.g., by regulating cellular metabolism).

This is an open access article under the CC BY license (http://creativecommons.org/licenses/by/4.0/).

Recent pan-genomic analyses of transcriptional regulation by hypoxia and binding of the HIF transcription factors to chromatin in cancer cell lines have highlighted the complexity of these responses, with many hundreds of genes under direct transcriptional regulation by HIF (Betts et al., 2013; Chi et al., 2006; Choudhry et al., 2014; Elvidge et al., 2006; Eustace et al., 2013; Ghazoui et al., 2011; Halle et al., 2012; Mole et al., 2009; Ortiz-Barahona et al., 2010; Schodel et al., 2013; Seigneuric et al., 2007; Smythies et al., 2019; Sorensen et al., 2010; Toustrup et al., 2011; Xia and Kung, 2009; Xia et al., 2009; Yang et al., 2018). However, different studies have identified non-overlapping sets of regulated genes in response to hypoxia. A meta-analysis of (largely) microarray assays of hypoxic gene regulation found that only a small number of genes were regulated in all studies (Ortiz-Barahona et al., 2010). However, the studies examined in this overview used heterogeneous experimental conditions encompassing varying degrees/durations of hypoxia, and it is not clear to what extent these differences affect the HIF target gene repertoire and hypoxic gene regulation. Other groups have attempted to define common hypoxia-induced genes by clustering gene expression patterns in microarray analysis of solid tumors with small sets of canonical hypoxia-regulated genes (Buffa et al., 2010; Hu et al., 2009; Winter et al., 2007) or with pimonidazole staining (Ragnum et al., 2015). However, studies often rely on previously defined gene signatures and have largely focused on only a few cancer types. Therefore, it is not clear to what extent they reflect hypoxic gene activation in other tumor types. Some of the most widely used hypoxia signatures were unable to distinguish constitutive activation of hypoxia pathways in ccRCC when applied to gene expression datasets in the large tumor databases TCGA (The Cancer Genome Atlas) and ICGC (International Cancer Genome Consortium) (Bhandari et al., 2019, 2020). Specifically, renal tumors (approximately 90% of which are ccRCCs) exhibited mid-range hypoxia scores relative to other tumor types in two studies; ranking 11th across 19 tumor types defined in one study (Bhandari et al., 2019) and ranking 14th across 27 tumor types in another (Bhandari et al., 2020). This suggests that the most commonly used hypoxia signatures reflect tissue-specific responses to hypoxia, which could be inherent to the models in which they were generated. These studies have been based solely upon transcriptional analyses and do not distinguish direct transcriptional targets of hypoxia pathways from indirect consequences. Recent studies have attempted to identify *bona fide* direct consequences of hypoxia on gene expression by combining transcriptomics approaches with analysis of HIF binding using chromatin immunoprecipitation sequencing (ChIP-seq) (Choudhry et al., 2014; Mole et al., 2009; Schodel et al., 2010; Xia and Kung, 2009; Xia et al., 2009). However, to date, these have been confined to relatively few cancer types, and their applicability across multiple tumor types has not been examined.

We therefore performed transcriptional profiling and whole-genome HIF binding assays (RNA sequencing [RNA-seq] and ChIP-seq) in NCBI cancer cell lines from 6 of the most common solid tumor types worldwide (WHO International Agency for Research on Cancer, 2020)—A549 cells (lung cancer), HCT116 cells (colorectal cancer), T47D cells (breast cancer), PC3 cells (prostate cancer), HepG2 cells (hepatocellular carcinoma), and RCC4 cells (kidney cancer)—to identify the direct transcriptional targets of HIF in each. However, ChIP-seq and RNA-seq analyses are restricted to the cell lines available and are biased against tumor types for which there are no cell line models, including rare cancer types or those that are not amenable to growth *in vitro*. Solid tumors are comprised of many (interacting) cell types, and these will not be captured in a cell monoculture. Even where such cell lines exist, they may not fully represent the heterogeneity seen across a range of different tumors. Just as non-malignant healthy cells often lose their physiological functions *in vitro*, tumor cells may also behave differently in culture than in their native environment. Tumor hypoxia often varies from region to region in the tumor, and this will not be adequately represented in monolayer culture. We therefore employed an integrated analysis of our 72 transcriptome and whole-genome binding datasets to define a core set of 48 HIF target genes that are conserved across multiple cell types. We then leveraged our core signature of HIF targets across 9,760 cancers in the TCGA bulk RNA-seq dataset to provide a pan-cancer analysis of HIF pathway activation across 32 different tumor types. We also show that these conserved HIF target genes can be applied to single-cell RNA-seq datasets from solid tumors and used to examine intra-tumor heterogeneity in HIF pathway activation in individual cancer samples and between different tumors. Using a panel of genes reduces the phenomenon of "dropout" typically observed in gene-level expression in single-cell datasets. Last, applying this gene signature to single-cell data allows the hypoxia response to be deconvoluted across diverse cell types in heterogeneous tumor samples. This includes not only identification of HIF activity in cancer cells but also in non-cancer cells in the tumor, allowing the effects of intra-tumor hypoxia on stromal responses (such as tumor angiogenesis) to be studied *in vivo* in solid tumors.

RESULTS

Analysis of HIF pathways in cell culture

First, RNA-seq assays were performed in triplicate in each cell line (a total of 36 RNA-seq datasets) after incubation in 21% oxygen (normoxia) or 0.5% oxygen (hypoxia) for 16 h (RCC4 cells were stably transfected with wild-type VHL to restore oxygen sensitivity to the HIF pathway) (Figure 1A). Immunoblot analysis confirmed induction of HIF-1α and HIF-2α in all 6 cell lines (Figure S1). Differentially expressed genes (adjusted $p < 0.05$, fold change > 1.2) were identified in each cell line (Figures 1A and S2A–S2F). There were approximately equal numbers of upregulated genes (average, 2,292 genes) and downregulated genes (average, 2,188 genes) in each cell line in hypoxia (Figure 1B). There were 6,604 genes upregulated by hypoxia and 6,356 genes downregulated by hypoxia in one or more cell lines. Of these, 47% (3,085 upregulated and 3,045 downregulated genes) were unique to a single cell line, only 1.8% (157 upregulated and 77 downregulated genes) were common to all 6 cell lines, and 6.6% (429 upregulated and 427 downregulated genes) were shared among 5 or more cell lines (Figure 1C).

We next examined HIF binding in each of the six cell lines using ChIP-seq with antibodies directed against HIF-1α, HIF-2α, and

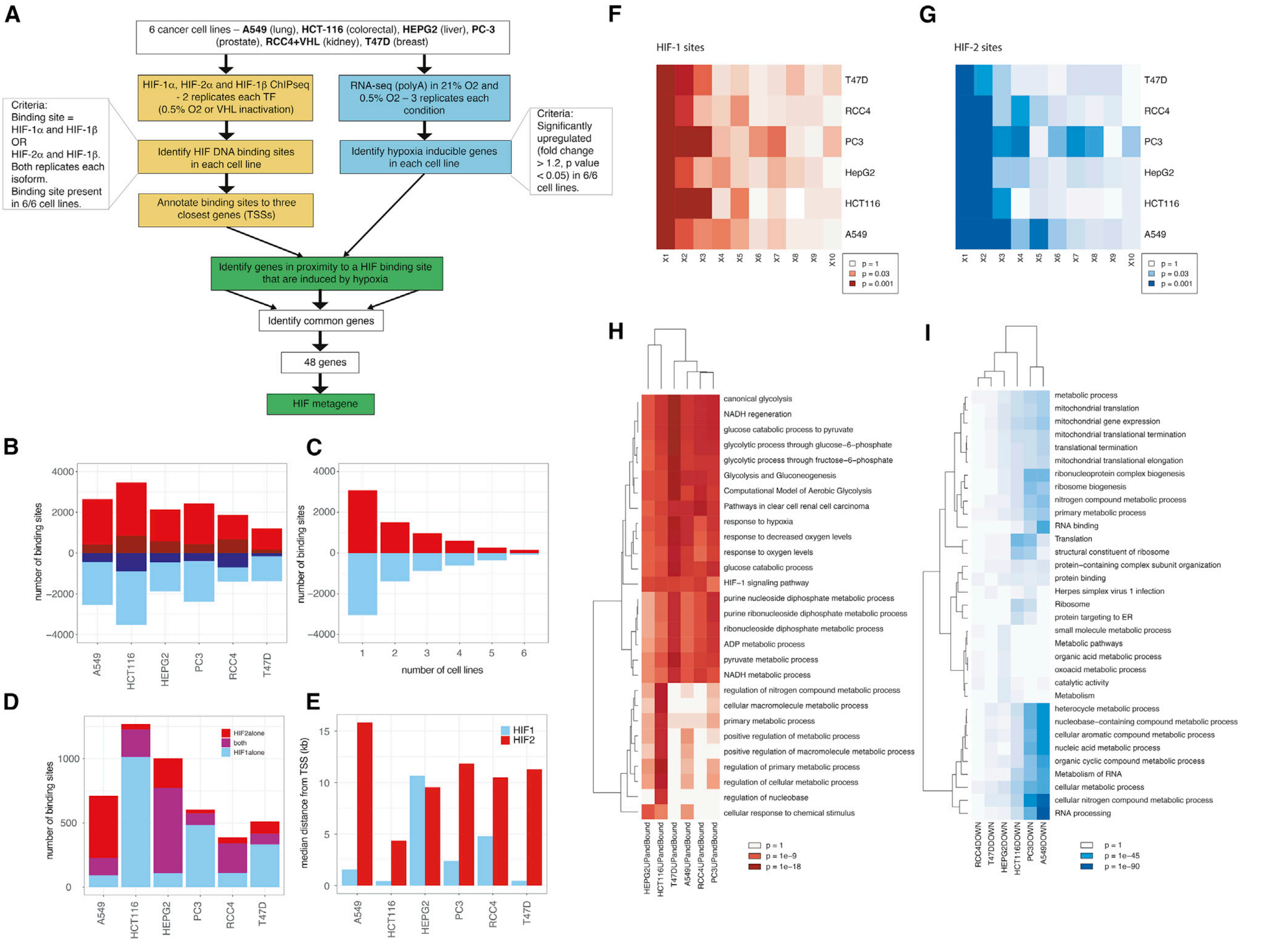

Figure 1. RNA-seq and ChIP-seq analysis of cancer cell lines

(A) Schematic of dataset analysis.

(B) the number of genes upregulated (red) or downregulated (blue) in each of the 6 cell lines. Adjusted $p < 0.05$, fold change ≥ 1.2 (those shown in dark red/blue are unique to that cell line).

(C) The number of genes upregulated (red) or downregulated (blue) in the specified number of cell lines.

(D) The number of canonical HIF-1 (blue), HIF-2 (red), or shared (purple) binding sites in each of the 6 cell lines.

(E) The median distance from the HIF-binding site to the nearest TSS for HIF-1 (blue) and HIF-2 (red).

(F) Heatmap showing p values for gene set enrichment analyses (GSEAs) for the first, second, third, etc. closest gene to each HIF-1 binding site among genes induced by hypoxia.

(G) The same heatmap for genes closest to HIF-2 binding sites.

(H) Gene Ontology biological pathway (GO:BP) analysis for genes upregulated and bound by HIF in each of the 6 cell lines using gProfiler.

(I) The same analysis for genes downregulated in each cell line.

HIF-1β (a total of 36 ChIP-seq datasets). High-confidence canonical HIF-1 and HIF-2 sites were defined based on ChIP-seq peaks called by MACS (model-based analysis of ChIP-seq) in both HIF-α and both HIF-1β ChIP-seq datasets. The total number of binding sites identified in each cell line ranged from 388 (RCC4 cells) to 1,269 (HCT116 cells) (Figure 1D), and although this broadly reflected the number of upregulated genes in each cell line ($r = 0.74$), with only 6 cell lines, the association did not reach statistical significance ($p = 0.09$). A549 cells had a higher proportion of HIF-2-bound sites, commensurate with relatively higher levels of HIF-2α protein in this cell line (Figures 1D and S1). HIF-α levels are a balance between production and degradation. Therefore, factors that alter the transcription and/or translation of each isoform in different cell types may further modify HIF-α levels and HIF target gene expression in hypoxia and after VHL inactivation. In each case, the canonical HRE motif RCGTG was the most enriched motif at HIF-1α- and HIF-2α-bound sites. In line with previous analyses, HIF-1 bound closer to the transcriptional start site of genes than HIF-2 (Figures 1E and S2G–S2R).

HIF binding was then correlated with gene regulation by hypoxia in each individual cell line using gene set enrichment

analysis (GSEA). In each cell line, the genes closest to each HIF binding site were enriched among genes upregulated in hypoxia but not among genes downregulated by hypoxia (Figures 1F, 1G, and S2S–S2X). This is consistent with HIF acting as an activator but not as a repressor. However, not all closest genes were upregulated in hypoxia. We therefore examined the second-closest gene to each HIF binding site and found that these were also enriched among hypoxia-induced genes (Figures 1F and 1G). In several cell lines, genes even more distant from HIF binding sites were enriched among hypoxia-induced genes. Taking this into account, to distinguish potential direct transcriptional targets of HIF binding, we identified genes that were upregulated by hypoxia (adjusted $p < 0.05$, fold change > 1.2) and one of the three closest genes to a HIF binding site in each of the cell lines. The number of HIF-bound and hypoxia-induced genes in each cell line ranged between 207 (T47D cells) and 673 (HCT116 cells). T47D cells having the lowest number of HIF-bound and hypoxia-upregulated genes was in line with consistently lower overall HIF-α protein levels in T47D cells (Figure S1). Biological pathways enriched among genes bound by HIF and upregulated by hypoxia in each cell line, using gProfiler, included glycolysis/gluconeogenesis, response to hypoxia, and ATP generation (Figure 1H). Genes downregulated in hypoxia were enriched for mitochondrial gene expression, ribosomal processes, protein translation, and cyclic compound metabolic processes (Figure 1I).

The majority (909 of 1,345 or 68%) of HIF-bound and upregulated genes were unique to a single cell line (Figures 2A and 2C). The number of genes shared between the different combinations of 2, 3, or 4 cells lines was comparatively small, and these were collectively under-represented (Figure 2B). However, genes that bound HIF and were upregulated by hypoxia in all 6 cell lines were over-represented (48 genes) (Figures 2A and 2B; Table 1). This strongly suggests the presence of a small core set of HIF-bound and hypoxia-upregulated genes with an increased likelihood of being shared in other cell lines. These genes were highly enriched for hypoxia pathways, glycolysis, and 2-OG (2-oxoglutarate)-dependent dioxygenases in gProfiler analysis. All 48 genes that were bound and upregulated in the 6 cell lines were bound by both HIF isoforms and so were identified in all 36 ChIP-seq datasets (Figure 2C). Each gene was upregulated in all 6 RNA-seq analyses (based on another 36 datasets). Therefore, these genes are likely to be extremely robust to statistical variation. Finally, these common genes were bound by HIF in considerably closer proximity to the transcriptional start site (TSS) (median distance 426 bp, $p = 6 \times 10^{-9}$, Wilcoxon rank-sum test) than at those genes that were bound and regulated in fewer cell lines or were unique to a single cell line (median distance, 12,226 bp) (Figure 2D). The cell-type-specific binding sites potentially represent HIF-bound enhancers (rather than HIF-bound promoters), which is consistent with enhancers often being promoter distal and cell type specific. At 4 of 48 gene loci, HIF had cell-type-specific binding sites that were in proximity to a common hypoxia-induced gene.

We next determined whether the core, consensus set of HIF-bound and hypoxia-upregulated genes identified in our 6 cell lines was induced by hypoxia and bound by HIF in cancer cell lines from an additional two, unrelated, common cancer types (cervical cancer: HeLa cells, GEO: GSE169041; melanoma: mel501 cells, GEO: GSE95280) (Louphrasitthiphol et al., 2019; Ortmann et al., 2021). In HeLa cells, all 48 genes were upregulated in hypoxia (Figure 2E) and bound by HIF (data not shown). Although only 41 of the 48 genes were detected in mel501 cells, 39 of these 41 were upregulated by hypoxia (Figure 2F). Thus, the core set of genes identified in our 6 cell lines defines a set of genes that are consistently hypoxia upregulated and HIF bound in other cancer cell lines. 43 of 48 genes were also induced by hypoxia in the human umbilical vein endothelial cell (HUVEC) cell line (GEO: GSE89840) (Tiana et al., 2018), indicating the applicability of this gene signature to non-cancerous and non-epithelial cells (Figure 2G). The hypoxia conditions used to generate HeLa, mel501, and HUVEC datasets differed from that used for our 6 cell line datasets; HeLa cells were exposed to 1% O_2 for 6 h, mel501 cells were exposed to 1% O_2 for 24 h, and HUVECs were exposed to 1% O_2 for 16 h. Therefore, these putative HIF target genes transcend the specific hypoxic conditions used in this study; thus, our gene signature can likely be applied to cells subjected to different durations of hypoxia.

Analysis of HIF pathways using TCGA RNA-seq datasets

We then examined whether our 48-gene signature similarly reflected HIF activation in solid tumors using TCGA RNA-seq data from 9,760 tumors across 32 different tumor types. This dataset includes RNA-seq analysis of 538 kidney renal clear cell carcinoma (KIRC) cancers. This tumor type is associated with a high prevalence of VHL mutation and constitutive activation of HIF. We initially combined the expression of the 48 genes into a single ‘‘HIF metagene’’ score that reflected their combined expression in each tumor. The expression of individual genes was first quantile normalized so that highly expressed genes would not dominate the signature before the scores for each gene were added. As predicted, clear cell renal cancers have the highest HIF metagene expression of any of the 32 tumor types (Figure 3A). This is in contradistinction to a previously defined hypoxia gene signature based on hypoxic gene expression in a limited number of cancer types that does not incorporate analysis of HIF binding (Bhandari et al., 2019, 2020).

We next compared HIF metagene expression in these tumor samples with that in normal solid tissue samples from the same TCGA projects. Clear cell renal cancers (KIRC) had significantly higher expression than normal tissue samples from the same project ($p < 2.2 \times 10^{-16}$), consistent with constitutive HIF activation resulting from loss of the VHL tumor suppressor gene (Figure 3B). Conversely, kidney renal papillary cell carcinoma and kidney chromophobe RCCs (KIRP and KICH) had only modest increases in the HIF metagene compared with normal tissue ($p = 0.0008$ and $p = 0.03$, respectively) (Figures 3C and 3D). This indicates that the HIF metagene can reliably detect HIF activation in the TCGA bulk RNA-seq datasets.

Even within a specific tumor type, individual samples displayed a wide-range of HIF-metagene scores (Figure 3A). To determine whether this reflected biological variation in HIF activation rather than simply statistical noise, we next examined whether the HIF metagene score correlated with other well-recognized HIF target genes within a single tumor type. Within

CellPress
OPEN ACCESS

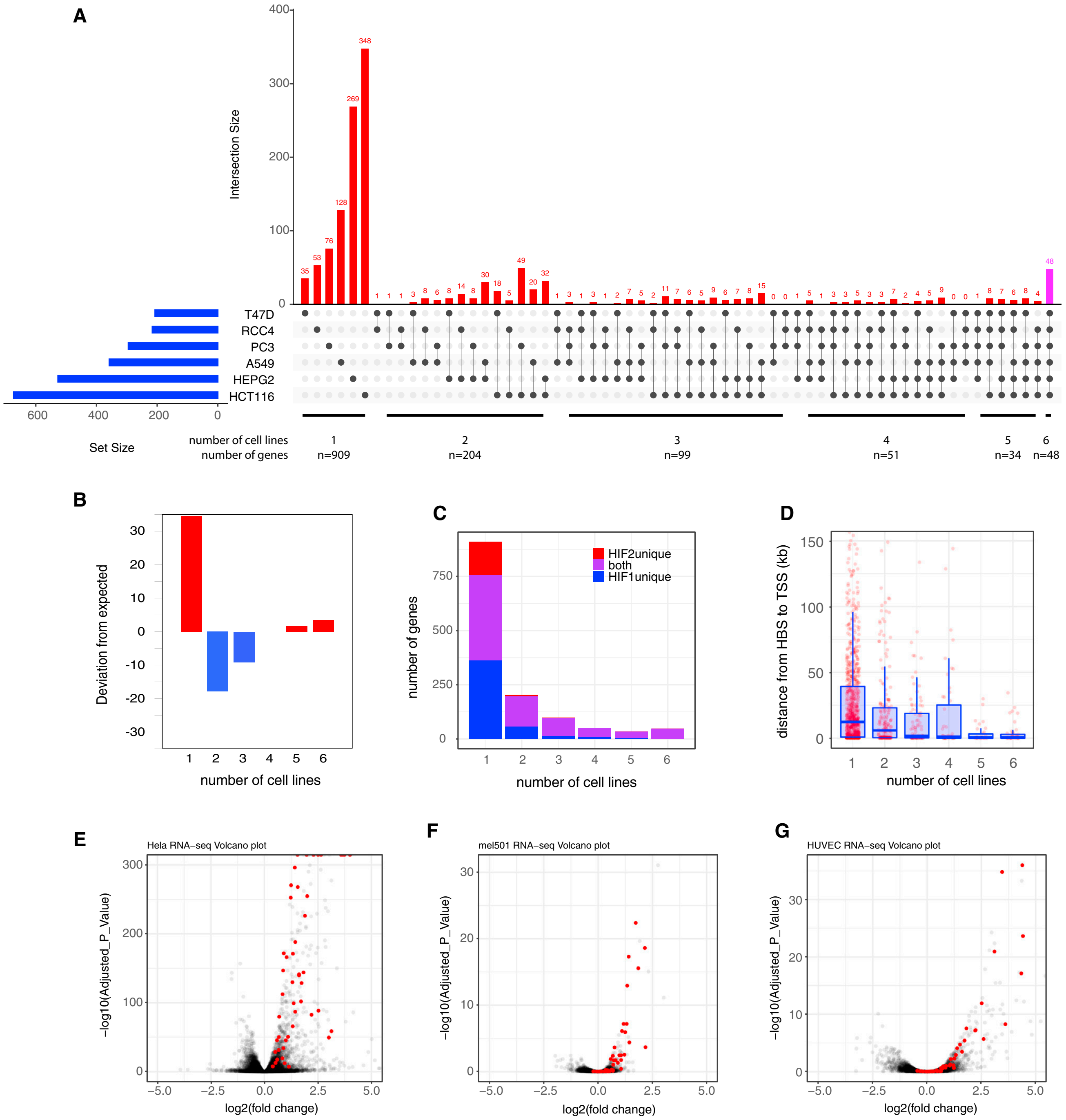

Figure 2. Overlap between HIF target genes in cancer cell lines

(A) UpSetR analysis of genes bound by HIF and upregulated in hypoxia in each of the 6 cell lines. The blue horizontal bars show the total number of HIF-bound, hypoxia-upregulated genes in each cell line. The red vertical bars show the number of genes common to the combinations of cell types denoted by the black dots as well as those unique to individual cell lines. The magenta vertical bar shows the number of genes common to all 6 cell types.

(B) Bar chart showing the deviation from expected in the number of genes common to different numbers of cell lines.

(C) Bar chart showing the number of genes bound by HIF-1, HIF-2, or both isoforms according to the number of cell lines in which they are bound and upregulated.

(D) Box-and-whisker plot showing the distance between HIF binding site (HBS) and transcriptional start site (TSS) for genes bound by HIF and upregulated under hypoxia in the denoted number of cell lines.

(E–G) Volcano plots showing −log10(adjusted p value) versus log2(fold change in RNA-seq analysis of gene expression in (E) HeLa cervical cancer cells, (F) mel501 melanoma cells, and (G) HUVECs (human umbilical vein cells) incubated under hypoxia versus normoxia.

Table 1. Conserved HIF target genes and their inclusion in other hypoxia mRNA signatures

	ADM	AK4	ALDOA	ANGPTL4	ANKRD37	BHLHE40	BNIP3L	C4orf3	C8orf58	CTDSP1	DDIT4	DENND1A	EGLN1	ENO1	ESYT2	FUT11	GBE1	GPI	HERC3	HK2	KCTD11	KDM3A	KDM4B	KDM4C
Benita														Y								Y		
Betts/Eustace			Y	Y										Y							Y			
Buffa	Y		Y		Y						Y			Y				Y		Y				
Elvidge	Y			Y			Y				Y						Y			Y		Y	Y	
Ghazoui					Y						Y			Y				Y						
Halle			Y					Y													Y			
Hu	Y			Y							Y													
Ortiz-Barahoma							Y						Y											
Ragnum	Y										Y													
Seigneuric				Y							Y			Y										
Sorensen	Y		Y		Y		Y						Y					Y		Y	Y	Y	Y	
Toustrup	Y		Y		Y		Y														Y			
Winter			Y	Y																	Y			
Yang						Y																		

	LDHA	LONP1	MIR210HG	MXI1	NCKIPSD	NDRG1	NPEPPS	P4HA1	PDLIM2	PFKFB4	PGAM1	PGK1	PKM	PLOD2	PNRC1	RBPJ	SAP30	SCD	SEMA4B	SLC16A3	SLC2A1	STC2	TNIP1	ZNF395
Benita								Y																
Betts/Eustace	Y							Y			Y	Y									Y			
Buffa	Y					Y		Y			Y	Y									Y			
Elvidge				Y		Y		Y				Y									Y	Y		
Ghazoui	Y					Y		Y			Y	Y								Y	Y			
Halle										Y												Y		
Hu						Y														Y				
Ortiz-Barahoma	Y					Y						Y												Y
Ragnum								Y										Y						
Seigneuric	Y			Y		Y		Y				Y						Y	Y					
Sorensen						Y		Y				Y		Y		Y	Y				Y			Y
Toustrup						Y		Y													Y			
Winter	Y					Y		Y		Y	Y	Y									Y			
Yang																								

the clear cell RCC (KIRC) cohort, the expression of several well-recognized HIF-target genes that did not comprise part of the HIF metagene was positively correlated with the metagene (Figures 4A–4D). We then extended this analysis to examine each of the genes that were previously identified as HIF bound and hypoxia induced in 5 of 6 cell lines but did not form part of the metagene itself. The correlation between the HIF metagene and each of these genes was determined in each of the 32 TCGA tumor-specific datasets (Figure 4E). Many of these genes correlated positively with the HIF metagene in multiple tumor-specific datasets, including the canonical HIF target genes *ALKBH5*, *BNIP3*, *EGLN3*, *GAPDH*, *P4HA2*, *PDK1*, *PFKL*, *PFKP*, *PLOD1*, and *TPI1*. This co-variation of the HIF metagene with other HIF target genes confirms an underlying biological variation in HIF metagene expression within each tumor type. This may arise from tumor-to-tumor differences or from differences in the region of each tumor sampled in the TCGA analysis.

We next examined whether genetic mutation within the tumors might account for some of this tumor-to-tumor variability in HIF metagene score. As expected, VHL copy number loss ($p = 2 \times 10^{-8}$) and *VHL* mutation status ($p = 0.006$) correlated with increased HIF metagene scores in clear cell kidney cancer biopsies (Figure S3). Mutation of *PBRM1* ($p = 0.0003$) and mutation of *mTOR* ($p = 0.005$) were also associated with increased HIF metagene scores in this tumor type, consistent with previous findings (Brugarolas et al., 2003; Gao et al., 2017; Hudson et al., 2002; Treins et al., 2002). The HIF metagene score was also associated with a number of other common gene mutations in non-renal cancer types (Figures S3B and S3C). In particular, *TP53*, *MUC16*, *PTEN*, *ARID1A*, and *TTN* mutations were each associated with altered HIF metagene levels in more than one tumor type. Thus, although differences in intra-tumor oxygen levels may also contribute to some of the observed variability in the HIF metagene, common tumor-associated genetic mutations also affect the HIF transcriptional output in different tumors.

We then leveraged this sample-to-sample variation in the HIF metagene to systematically examine additional genes that co-vary with the HIF metagene in each tumor type. A systematic approach was employed to identify HIF metagene-associated genes in each of the 32 tumor types by testing each of the approximately 50,000 genes individually for correlation with the HIF-metagene in each tumor type dataset (Table S1). A significance threshold of $p \leq 1 \times 10^{-6}$ was used to allow multiple gene comparisons, and the 48 genes that contribute to the HIF metagene were excluded. In 12 of the tumor types (adrenocortical carcinoma - ACC, cholangiocarcinoma - CHOL, diffuse large B-cell lymphoma - DLBC, glioblastoma - GBM, kidney chromophobe - KICH, mesothelioma - MESO, ovarian serous cystadenocarcinoma - OV, rectum adenocarcinoma - READ, sarcoma SARC, skin cutaneous melanoma - SKCM, uterine carcinosarcoma - UCS, and uveal melanoma - UVM), very few genes reached statistical significance, likely because of the small numbers of tumors in each of these datasets underpowering these analyses (Figure 4F). However, overall, in each cancer type, a median of 698 genes correlated positively with the HIF metagene, and a median of 484 genes correlated negatively with the HIF metagene. Most of these genes were specific to either one or just a few cancer types; in total, 6,863 (54.8%) of the HIF metagene positively correlated genes were identified in a single cancer type, 2,467 (19.7%) correlated in two cancer types, and 1,181 (9.4%) correlated in three cancer types (Figure 4G). This is similar to the pattern observed in the cell culture models, in which most HIF-target genes were cell type specific. However, 168 genes were positively correlated with the HIF metagene in more than 10 cancer types (62 genes in 15 or more cancer types and 23 genes in 20 or more cancer types). These genes were strongly enriched for hypoxia/HIF and carbohydrate metabolism pathways and included a number of well-described canonical HIF target genes, such as *CA9*, *EGLN3*, *BNIP3*, and *VEGFA*, that are not in our cell-line-derived HIF metagene. This validates our correlative approach of leveraging

CellPress
OPEN ACCESS

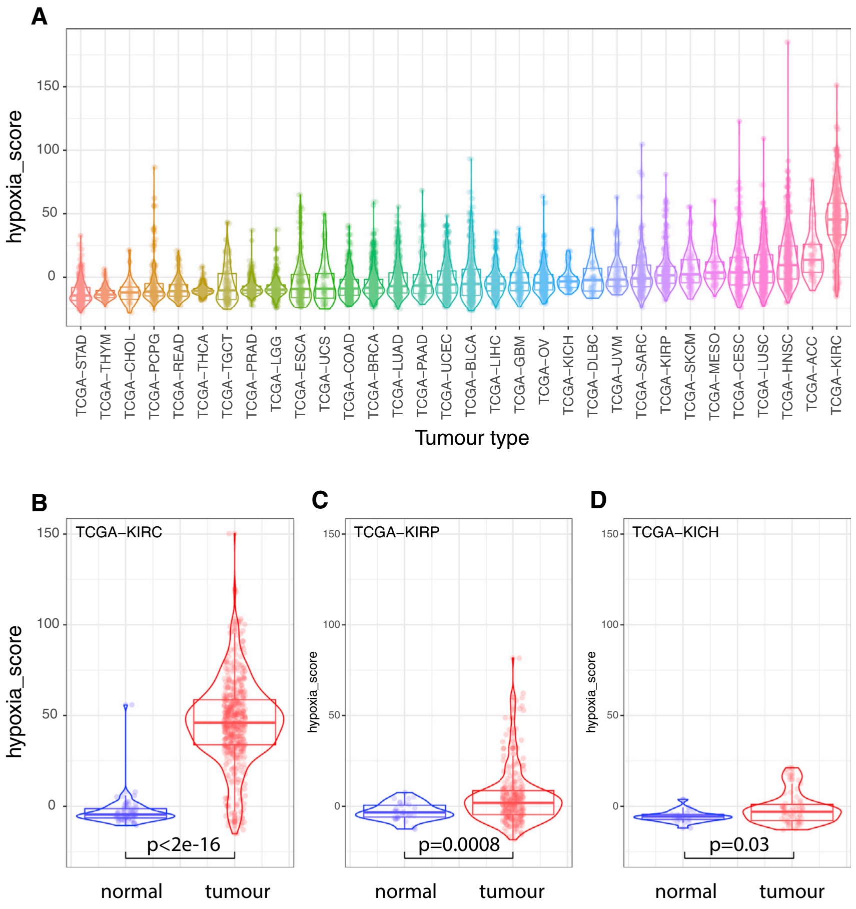

Figure 3. The HIF metagene identifies HIF activation in solid tumors

(A) HIF metagene expression in RNA-seq analysis of 9,760 tumors from the TCGA database. Tumors are grouped according to tumor type and ranked according to median expression for that tumor type.

(B–D) HIF metagene expression in RNA-seq analysis of tumors and normal tissue for (B) clear cell RCC (TCGA-KIRC), (C) papillary RCC (TCGA-KIRP), and (D) chromophobe RCC (TCGA-KICH). The p values are for Wilcoxon rank-sum test.

the HIF metagene to identify additional HIF associated genes from the TCGA RNA-seq analyses.

Similarly, genes negatively correlated with the HIF metagene were also highly specific to one or just a few cancer types; in total, 6,704 (58.1%) of the HIF metagene negatively correlated genes were identified in a single cancer type, 2,968 (21%) correlated in two cancer types, and 1,272 (9%) correlated in three cancer types. Only 8 genes were negatively correlated in more than 10 cancer types, although 179 genes were negatively correlated in 7 or more and were enriched for transcriptional regulation pathways. Because HIF activates (and does not repress) its direct transcriptional targets, the genes that negatively correlate with the HIF metagene will reflect genes indirectly downregulated upon HIF activation. Therefore, not only can our HIF metagene be utilized to study tissue-specific HIF target genes, but it can also be used to identify the indirect consequences of HIF activation on gene expression.

We next examined the genes that positively correlated with the HIF metagene in individual TCGA tumor types for enrichment among Gene Ontology biological pathways (GO:BP) using gProfiler (Figure S4A). For each tumor type in which more than 200 genes correlated, the 200 genes with the strongest correlation were analyzed. As expected, pathways involved in cellular responses to hypoxia and carbohydrate metabolism were commonly enriched among positively correlated genes in many of the tumor types. Genes involved in epithelial development, keratinization, and cornification were enriched among HIF metagene-associated genes in stomach adenocarcinoma (STAD), esophageal (ESCA), bladder urothelial carcinoma (BLCA), and head and neck squamous cell carcinoma (HNSC) tumors. Genes involved in cell death were also enriched among positively correlated genes in these tumor types as well as in cervical squamous cell carcinoma and endocervical adenocarcinoma (CESC), sarcoma (SARC), lung squamous cell carcinoma (LUSC), and glioblastoma multiforme (GBM) tumors. Genes involved in copper metabolism are positively correlated with the HIF metagene in testicular germ cell tumors (TGCTs), whereas those involved in exocytosis pathways are enriched for HIF metagene-associated genes in thyroid carcinoma (THCA) tumors. Finally, angiogenic pathway genes are particularly enriched among HIF metagene-associated genes in papillary renal (KIRP) tumors.

Conversely, genes involved in regulation of transcription were negatively correlated with the HIF metagene in testicular germ cell tumors (TGCT), HNSC, uterine corpus endometrial carcinoma (UCEC), thymoma (THYM), THCA, and liver hepatocellular carcinoma (LIHC) tumors (Figure S4B). Similarly, genes negatively correlated with the HIF metagene in breast invasive carcinoma (BRCA) and LUSC tumors were enriched for roles in the immune response, and genes negatively correlated in brain lower grade glioma (LGG) tumors were enriched for roles in DNA repair, DNA replication, and cell cycling.

Analysis of HIF pathways in scRNA-seq datasets

We next examined whether the HIF metagene derived from the 6 cancer cell lines might also be applied to single-cell RNA-seq (scRNA-seq) datasets. As an initial test, scRNA-seq was performed on 2,926 cells from primary patient-derived cell cultures from 2 regions within ccRCC and from the surrounding (normal) kidney. Cultures were incubated in 21% oxygen (normoxia) or 0.5% oxygen (hypoxia) for 16 h before being subjected to 3′ scRNA-seq on the 10X Genomics platform. Cells from the two tumor samples formed clusters separate from normal renal cells.

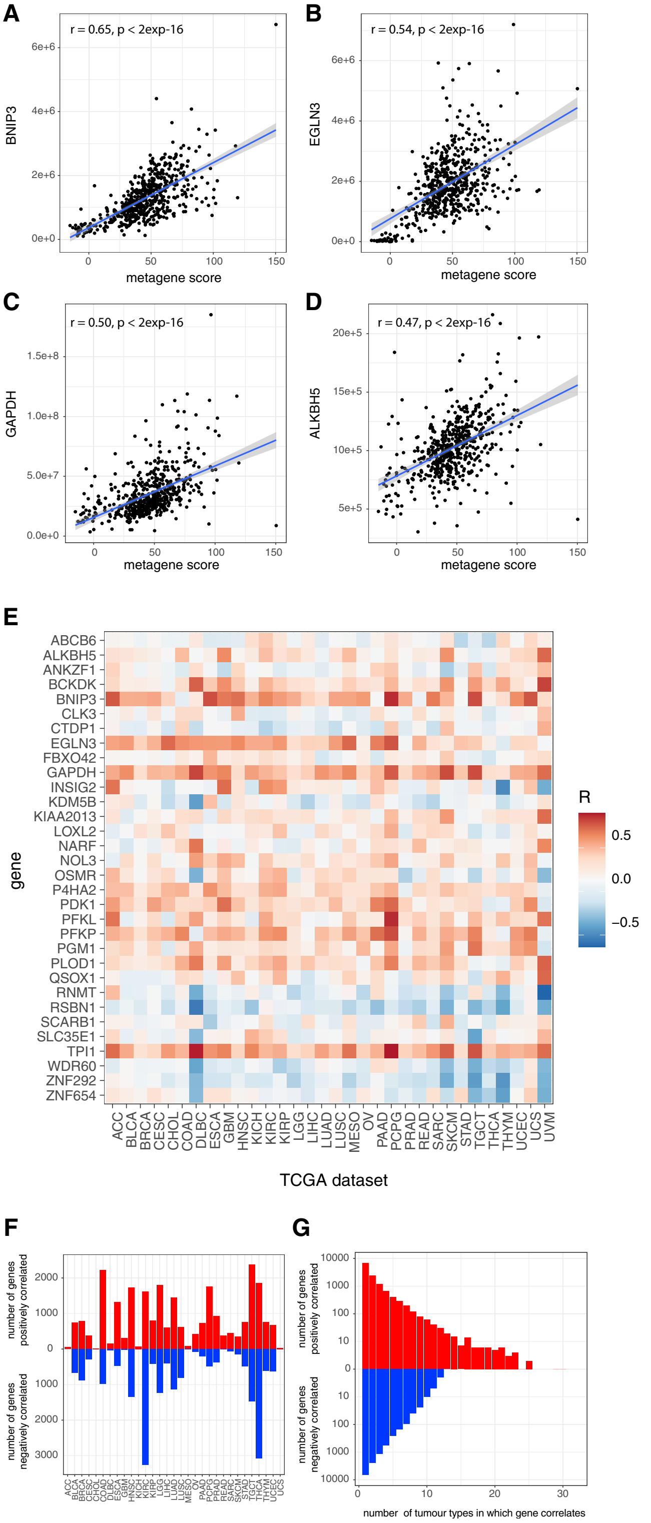

Figure 4. Analysis of genes correlated with the HIF metagene in the TCGA tumor RNA-seq datasets

(A–D) Scatterplots showing the Pearson correlation between the HIF metagene and individual canonical HIF target genes in the TCGA-KIRC dataset.

(E) Heatmap showing the correlation coefficient between the HIF metagene and individual genes (those bound and regulated in 5 of 6 cell lines but not included in the metagene) in each of the 32 TCGA tumor types.

(F) Bar chart showing the number of positively and negatively correlated genes ($p \leq 1 \times 10^{-6}$) in each of the TCGA datasets.

(G) Bar chart showing the number of positively and negatively correlated genes ($p \leq 1 \times 10^{-6}$) shared between multiple TCGA datasets.

Normal cells formed several subclusters representing different cell types, each of which included cells from the normoxic and hypoxic cultures (Figure 5A). This indicates that changes in gene expression resulting from hypoxia are minor compared with those that distinguish different cell types and that hypoxia does not interfere with the ability to distinguish cell identity in scRNA-seq analysis. Compared with normal renal cells, cells from the two tumor samples had high levels of the HIF metagene ($p < 2 \times 10^{-16}$, Wilcoxon rank-sum test), consistent with inactivation of VHL and constitutive stabilization of HIF in tumor cells (Figures 5B and 5C). Normal renal cells incubated under normoxia had low levels of the HIF metagene, and normal cells incubated under hypoxia had intermediate levels of the HIF metagene (Figure 5C). Hypoxic induction of the HIF metagene was observed in all cell clusters from normal renal cultures, including those representing epithelial and non-epithelial cells (Figure S5). This indicates that the HIF metagene can act as a marker of cell hypoxia in scRNA-seq analysis for multiple cell types and across cell lineages. Because hypoxic normal cells do not cluster distinctly from normoxic normal cells, this indicates that unsupervised clustering algorithms cannot distinguish cells subject to acute hypoxia and highlights the need for a HIF metagene to identify these cells.

We next examined whether this approach could also be applied to scRNA-seq analysis of freshly dissociated cells from tissue samples, which more closely reflect *in vivo* oxygenation and include more diverse cell types that do not grow well in culture. Because tissue samples may become ischemic *ex vivo*, we first examined whether sample storage might confound HIF metagene expression in scRNA-seq analyses. Data for 239,226 cells derived from lung, esophagus, and spleen samples stored at 4°C for 0, 12, 24, or 72 h prior to analysis were downloaded from https://www.tissuestabilitycellatlas.org (Madissoon et al., 2019), and HIF metagene scores were generated for each cell. Overall, HIF metagene scores correlated poorly with cold ischemia time in each tissue (Figure S6), with no consistent trend observed across tissue types (r = 0.049, r = −0.055, and r = −0.13, respectively). This indicates that HIF metagene scores are robust to delays in biospecimen handling.

Data for 13,213 normal and tumor cells from untreated patients with clear cell kidney cancer (Krishna et al., 2021) were then downloaded from https://trace.ncbi.nlm.nih.gov/Traces/index.html?view=analysis&acc=SRZ190804. ccRCC cells are frequently characterized by chromosomal copy number alterations (CNAs). These often encompass loss of chromosome 3p, resulting in inactivation of one VHL allele (the other allele generally being inactivated through mutation). InferCNV was therefore used to identify copy number abnormalities present in the tumor cell population, using the 3,110 normal cells as a reference (Figure 5D). Of the 10,103 cells in the tumor sample, 5,395 (53%) had loss of

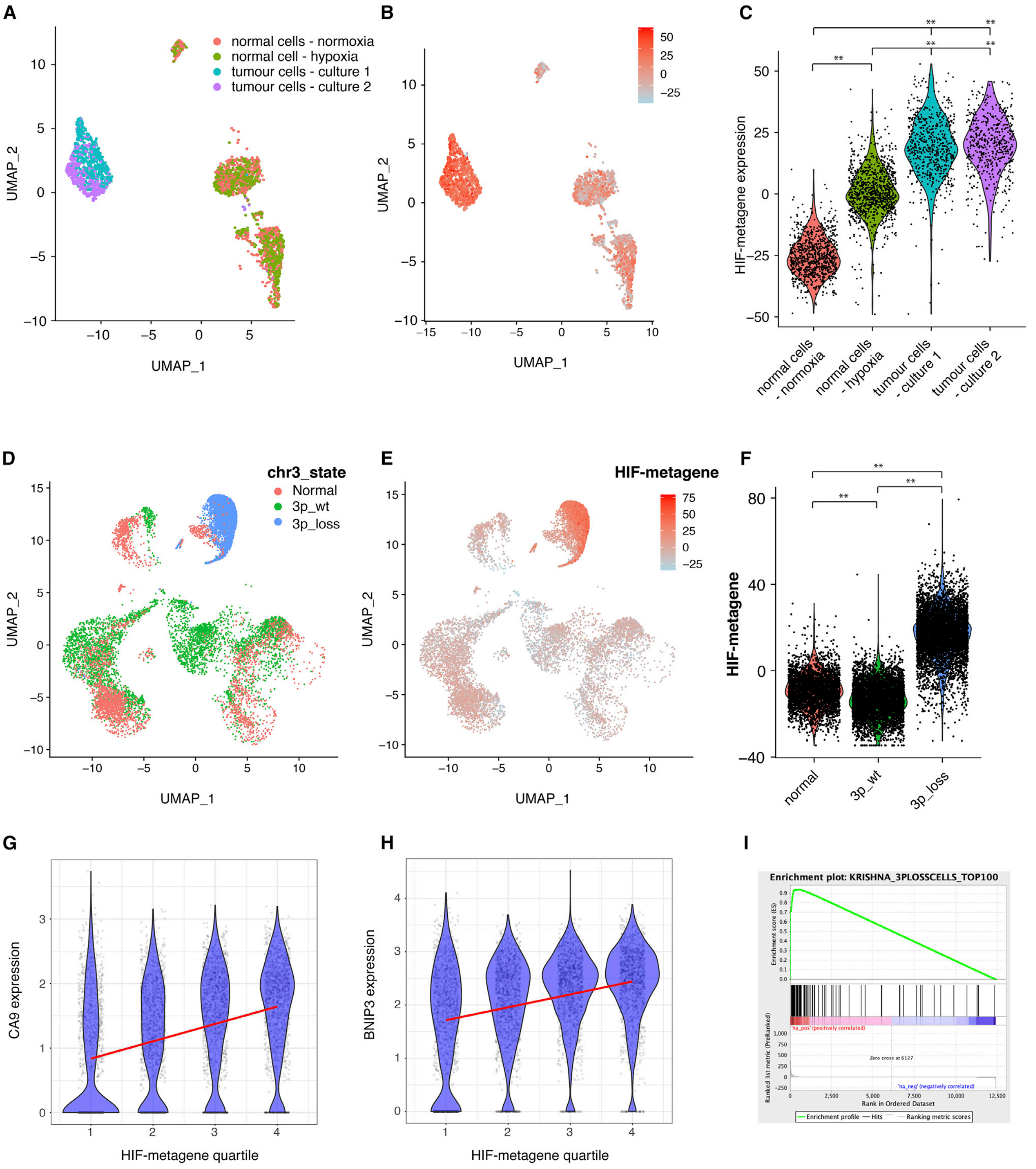

Figure 5. The HIF metagene identifies HIF activation in scRNA-seq analysis

(A–C) scRNA-seq analysis of cultured ccRCC cells and mixed population of normal kidney cells (incubated under normoxia and 0.5% hypoxia for 16 h). Shown are UMAP (uniform manifold approximation and projection) plots with (A) cells colored according to sample and (B) cells colored according to HIF metagene expression and (C) a violin plot showing HIF metagene expression in cultured mixed normal kidney cells incubated under normoxia and hypoxia and in ccRCC cells (**$p < 10^{-16}$, Wilcoxon rank-sum test).

(D–I) scRNA-seq analysis of freshly dissociated kidney and ccRCC samples.

(legend continued on next page)

chromosome 3p (encompassing the VHL locus) and formed a distinct cluster previously characterized as malignant cells by the authors based on marker gene expression. The remaining cells had no chromosomal abnormalities and have been previously annotated as immune and stromal cells in the same way (Krishna et al., 2021). Cells from normal kidney samples and chromosomally normal cells from the tumor samples had comparable levels of HIF metagene expression (Figures 5E and 5F). However, tumor cells with loss of chromosome 3p had significantly higher levels of HIF metagene expression ($p < 2 \times 10^{-16}$, Wilcoxon rank-sum test) than cells from the normal kidney sample or chromosomally normal stromal and immune cells from the tumor sample (Figures 5E and 5F). This confirms that the HIF metagene can identify HIF activation in ccRCC cells in scRNA-seq analysis of freshly excised tissue. Because 48 individual genes contribute to the HIF metagene, very few cells exhibit the phenomenon of "dropout," where no expression is detected for any of the 48 genes. Therefore, information about the state of HIF activation can be obtained from the majority of cells. However, the HIF metagene score varied widely between individual cells, even within the tumor cell (3p_loss) cluster. To determine whether this reflected biological variation in HIF activation between cells or simply stochastic differences in gene measurement, we again examined the correlation between the HIF metagene and other canonical HIF target genes that did not contribute to the metagene. CA9 and BNIP3 correlated positively with the HIF metagene ($p < 10^{-16}$) in multiple regression analysis, even after potential confounding variables, such as the number of counts and features per cell, were taken into account (Figures 5G and 5H). This suggests that at least some of the variation in the HIF metagene in the tumor cells is due to different levels of HIF activation and that the HIF metagene can be used to distinguish cells in a specific cluster that have differing levels of HIF activation. Finally, we tested whether the 100 genes correlating most strongly with the HIF metagene in single ccRCC malignant cells (defined by chromosome 3p loss) were regulated by hypoxia in the VHL-recomplemented ccRCC cell line RCC4+VHL. Genes correlating with the HIF metagene in scRNA-seq analyses were highly enriched for genes that were induced by hypoxia in our cell line bulk RNA-seq analyses, confirming the validity of this approach (Figure 5I).

Because the HIF-metagene was derived from a core consensus set of HIF-bound and hypoxia-induced genes in cell lines from 6 diverse cancer types, we next examined whether it also reflected HIF activation in non-ccRCC tumors, in which HIF is regulated by intra-tumor hypoxia. Data for 178,441 normal and tumor cells from 44 patients with early and late-stage lung adenocarcinoma (Kim et al., 2020) were downloaded from GEO (GEO: GSE131907). The HIF metagene score was significantly increased in early- and late-stage adenocarcinoma cells compared with normal alveolar epithelial cells (Figure 6A). Malignant cells from late-stage tumors (tL/B) had a higher HIF metagene score than those from early-stage tumors (tLung). Malignant cells from brain (mBrain) and lymph node metastases (mLN) also had an elevated HIF metagene score comparable with that of malignant cells from late-stage primary tumors (tL/B). This is consistent with a more hypoxic microenvironment in tumors than in normal lung tissue or possible HIF activation by other oncogenic pathways. However, when we extended the analysis to non-malignant cells within the tumors, we observed that endothelial cells from early-stage tumors and from brain metastases had a significantly elevated HIF metagene score compared with endothelial cells from normal lung tissue (Figure 6B), concordant with the differences observed in normal epithelial/malignant cells. This indicates that the HIF metagene can be used to determine levels of hypoxia in non-malignant stromal cells in the tumor. Because endothelial cells do not harbor oncogenic mutations, it suggests that the observed HIF activation in lung adenocarcinoma cells results from intra-tumor hypoxia rather than oncogenic HIF activation. There were insufficient numbers of endothelial cells from late-stage tumors and from mLN to determine differences in these populations. However, because each of these tumor categories comprises samples from different individuals, we next examined whether the HIF metagene could be used to determine differing levels of hypoxia in early-stage tumors. Tumor cells from samples T18, T20, T28, T31, and T34 had higher HIF metagene scores than the other 6 samples (Figures S7A and S7B). This pattern was again mirrored in non-cancer cell populations from the same samples, which do not harbor oncogenic mutations, so that the mean HIF metagene score for cancer cells from each sample positively correlated with that for non-cancer cells ($p = 0.02$). This correlation again indicates that the differences between samples result, at least in part, from differing degrees of intra-tumor hypoxia rather than random variation.

HIF metagene scores varied widely within the malignant cell population and within the endothelial cell population. We therefore examined whether this variation within cell populations correlated with other canonical HIF target genes that were not included in the HIF metagene. For both cell populations, several canonical HIF target genes showed highly significant correlation with the HIF metagene, including *GAPDH* (Figures 6C and 6D). The 100 genes that correlated most strongly with the HIF-metagene in malignant cells were significantly enriched among genes induced by hypoxia in our RNA-seq analysis of the A549 lung adenocarcinoma cell line (Figure 6E). Similarly, the 100 genes that correlated most strongly with the HIF metagene in the endothelial cell population were enriched among genes induced

(D) UMAP plot showing single-cell chromosome 3p copy number variation in cells extracted from an untreated clear cell kidney cancer and from surrounding normal kidney (from Krishna et al., 2021).
(E) UMAP plot showing HIF metagene expression in the same cells.
(F) Violin plot showing HIF metagene expression in normal cells and stromal and immune cells from the tumor (characterized by normal chr3 copy number) and tumor cells (characterized by chr3p loss) (**$p < 2 \times 10^{-16}$, Wilcoxon rank-sum test).
(G and H) correlation between the HIF metagene and canonical HIF target genes in tumor cells with chromosome 3p loss.
(I) GSEA of the top 100 genes correlating with the HIF metagene in cells with chr3p loss, showing enrichment among genes upregulated by hypoxia in the ccRCC cell line RCC4+VHL (ES = 0.94, NES = 1.39, p = 0.003). ES - enrichment score, NES - normalised enrichment score.

CellPress
OPEN ACCESS

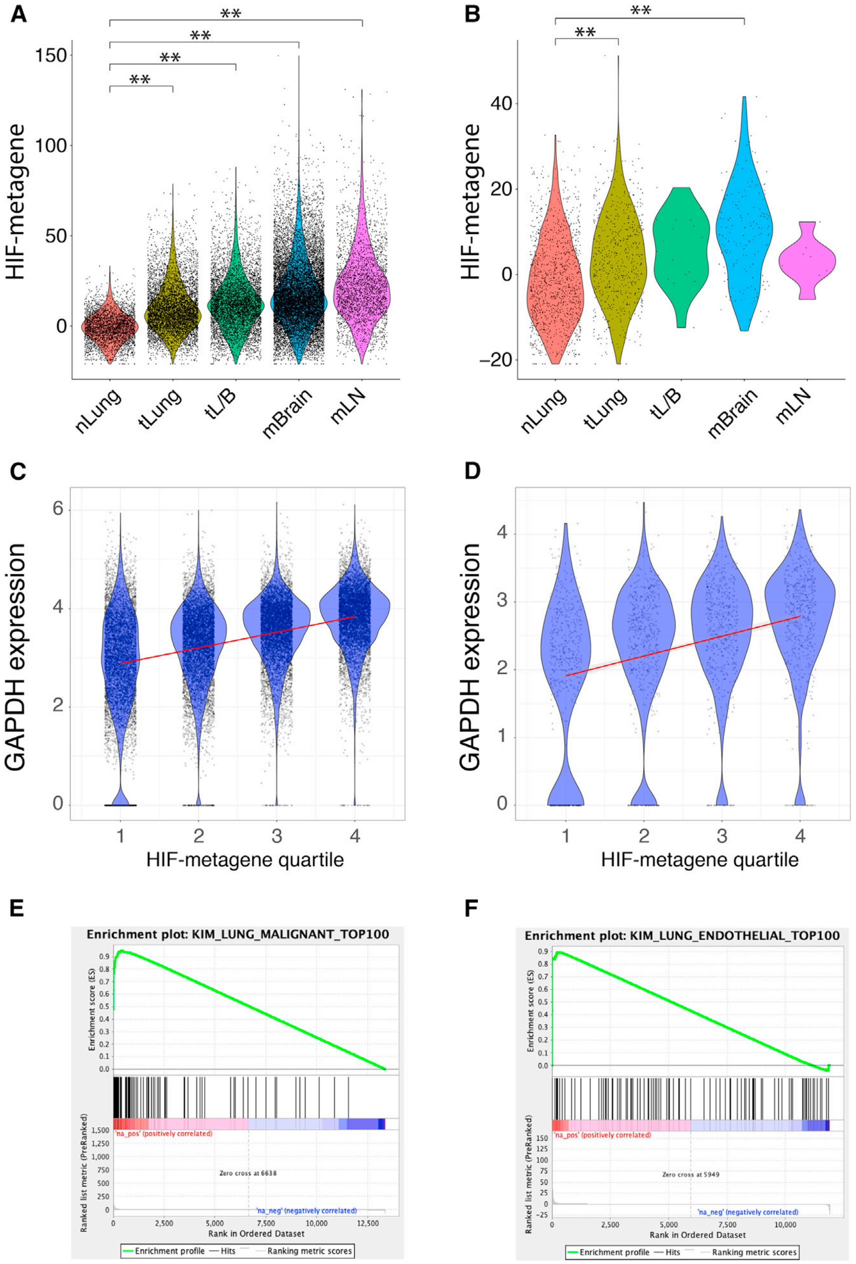

Figure 6. The HIF metagene identifies HIF activation in non-ccRCC tumors

(A and B) HIF-metagene score in (A) epithelial/tumor cells and (B) endothelial cells from normal lung (nLung), early-stage lung adenocarcinoma (tLung), late-stage lung adenocarcinoma (tL/B), and brain (mBrain) and lymph node metastases (mLN) (data from Kim et al., 2020) (**$p < 2 \times 10^{-16}$, Wilcoxon rank-sum test).

(C and D) Correlation between the canonical HIF target gene GAPDH and HIF metagene score in (C) malignant and (D) endothelial cells. Cells were first separated into quartiles according to HIF metagene score, and the GAPDH signal for each quartile is shown by violin plot and scatterplot.

(E) The top 100 genes that correlated with the HIF metagene in adenocarcinoma cells are enriched among genes induced by hypoxia in the A549 adenocarcinoma cell line (ES = 0.95, NES = 1.66, p = 0.005).

(F) The top 100 genes that correlated with the HIF metagene in endothelial cells are enriched among genes induced by hypoxia in the HUVEC line (ES = 0.89, NES = 1.30, p = 0.11).

by hypoxia in RNA-seq analysis of the HUVEC cell line (Tiana et al., 2018) (GEO: GSE89831), although this did not reach statistical significance (Figure 6F). Similarly, many of the genes that were identified as HIF target genes in 5 of 6 cancer cell lines positively correlated with the HIF metagene in malignant cells from each individual early-stage lung cancer sample (Figure S7C). This indicates that the HIF metagene can be used to determine hypoxic gene activation between and within populations of malignant and non-malignant tumor cells and in individual tumor samples from diverse cancer types. This can include tumor and non-tumor cell types for which there are no cell line data.

DISCUSSION

We combined transcript analysis with ChIP-seq in multiple cell types (representing breast, prostate, lung, colon, kidney, and liver cancer) exposed to hypoxia under uniform conditions to derive a HIF metagene signature. By combining HIF binding with hypoxic mRNA regulation, this analysis distinguishes the direct transcriptional response to hypoxia orchestrated by HIF from indirect effects. In all cell lines, HIF binding was associated with upregulated but not downregulated genes, indicating that HIF acts as a transcriptional activator but not as a repressor. The number of HIF target genes shared by all 6 cell lines was greater than expected from the overlap between lesser numbers of cell lines. These HIF target genes are conserved as hypoxia-responsive genes in RNA-seq analyses of cancer, non-cancer, epithelial, and non-epithelial cell lines. Therefore, this 48-gene set represents a core, consensus set of HIF targets genes that transcends the cell types in which the gene signature was defined.

We then demonstrate the applicability of a HIF metagene signature based on this gene set to human tumor data (TCGA) and to single-cell analyses of cultured and freshly derived cells. Within the scRNA-seq analysis, assessing the combined expression of the 48 core HIF target genes allows a HIF metagene score to be assigned to the vast majority of individual cells. This circumvents the phenomenon of "dropout," which applies when examining single genes in scRNA-seq data.

Importantly, we validated our signature against other measures of hypoxic and oncogenic HIF activation at the tumor level and for single cells. Unlike a number of other commonly used hypoxia gene signatures (Figure S8), our HIF metagene correctly identifies HIF activation in VHL-defective ccRCC in bulk RNA-seq analysis of TCGA tumors and in scRNA-seq analysis of individual cells. In the bulk RNA-seq analysis of TCGA tumors, there is considerable variation between HIF metagene scores even between tumors of the same type. This variation correlates with other well-described HIF target genes that are not part of our signature, indicating that it represents biological variation in HIF activation between tumors. We then leverage this variability to identify additional positively and negatively HIF-associated genes in each tumor type. This allows us to identify novel and tissue-specific HIF-associated genes even in tumor types for which there are no cell line data. Similar variability in HIF metagene scores is also observed between individual cells in the scRNA-seq analyses, and again this correlates with other well-described HIF target genes, indicating that it can identify biological variation in HIF activation between individual cells. Again, this variability can be leveraged to identify novel HIF-associated genes in diverse cell types within the tumor. Even in a solitary sample, this scRNA-seq analysis can be applied to deconvolute cell-type-specific transcriptional responses to HIF activation. This allows other sources of variability to be minimized (by comparing cells in the same sample) and demonstrates the applicability of this strategy even to scRNA-seq datasets with a small sample size.

An exploration of the underlying causes of this observed biological variability revealed that the HIF metagene is able to distinguish oncogenic and microenvironmental influences on HIF activation. First, in the TCGA bulk RNA-seq analysis of ccRCC, the HIF metagene score correlated with mutation in *PBRM1* (polybromo 1) and *mTOR* (mechanistic target of rapamycin kinase), both of which have been associated previously with HIF activation (Brugarolas et al., 2003; Gao et al., 2017; Hudson et al., 2002; Treins et al., 2002). CNA on chromosome 2 (on which the *EPAS1* gene encoding the HIF-2α isoform resides) was also correlated with the HIF metagene. Oncogenic mutation in other tumor types also correlated significantly with HIF activation, most commonly *TP53*, *MUC16*, *PTEN*, *ARID1A*, and *TTN*. This indicates that oncogenic mutations beyond those in the VHL gene are associated with altered HIF activation across a wide range of tumor types. Second, in scRNA-seq analysis of cells from multiple lung adenocarcinomas, we observed a correlation between HIF metagene score in adenocarcinoma cells and stromal cells. Because stromal cells do not harbor oncogenic mutations, this must therefore represent HIF activation as a consequence of microenvironmental factors and/or paracrine signaling from tumor cells. Thus, in addition to oncogenic influences on HIF activity, the HIF metagene can distinguish *in vivo* responses to microenvironmental hypoxia. This can be observed in cancer cells and in multiple specific stromal cell subtypes, allowing the tissue-orchestrated response to hypoxia to be deconvoluted at the single-cell level.

Limitations of the study

Our analysis aims to define a set of commonly (rather than universally) HIF-regulated genes because it is impossible to analyze every cell type. Genes may be lost from the list of common genes when they fail to reach any one of several stringent statistical thresholds in any of the cell lines studied. Our metagene does represent a set of genes, the majority of which will be HIF target genes in all cell types. A combined score based on these genes will therefore represent HIF activation in most cell types. Reducing the number of genes will make it less likely that the HIF metagene will reflect HIF activation in each cell line. Conversely, increasing the number of genes by relaxing the criteria may increase the proportion of non-regulated genes in any one cell type. This would dilute the ability of the metagene to distinguish HIF activation from stochastic noise.

STAR★METHODS

Detailed methods are provided in the online version of this paper and include the following:

- KEY RESOURCES TABLE
- RESOURCE AVAILABILITY
  - Lead contact
  - Materials availability
  - Data and code availability
- EXPERIMENTAL MODEL AND SUBJECT DETAILS
  - Human cell lines
  - Primary cell cultures
- METHOD DETAILS
  - Western blotting
  - ChIP-seq
  - ChIP-seq analysis
  - Bulk RNA-seq
  - Bulk RNA-seq analysis
  - Single cell RNA-seq
  - Single cell RNA-seq analysis
  - Gene set analysis
  - TCGA RNA-seq data
  - Sequencing
- QUANTIFICATION AND STATISTICAL ANALYSIS

SUPPLEMENTAL INFORMATION

Supplemental information can be found online at https://doi.org/10.1016/j.celrep.2022.111652.

ACKNOWLEDGMENTS

This study was supported by the National Institute for Health Research (NIHR-RP-2016-06-004 to D.R.M.); Cancer Research UK (A416016 to D.R.M.); the Deanship of Scientific Research, King Abdulaziz University, Ministry of High Education for Saudi Arabia (to H.C., D.R.M., and P.J.R.); the Ludwig Institute for Cancer Research (to P.J.R.); the Wellcome Trust (106241/Z/14/Z to P.J.R.); and the Francis Crick Institute (to P.J.R.), which receives its core funding from Cancer Research UK (FC001501), the UK Medical Research Council (FC001501), and the Wellcome Trust (FC001501). The computational aspects of this research were supported by Wellcome Trust Core Award 203141/Z/16/Z and the NIHR Oxford BRC. The views expressed are those of the author(s) and not necessarily those of the NHS, the NIHR, or the Department of Health. We are grateful to Lisa Browning and the Oxford Radcliffe Biobank for assisting with acquisition of freshly excised ccRCC tissue and tumor-adjacent normal kidney for dissociation.

AUTHOR CONTRIBUTIONS

Conceptualization, O.L., H.C., P.J.R., and D.R.M.; methodology, O.L., R.L., S.H., and D.R.M.; software, R.L., S.H., and D.R.M.; formal analysis, R.L., S.H., and D.R.M.; investigation, O.L., R.L., S.H., and D.R.M.; data curation, R.L., S.H., and D.R.M.; writing – original draft, O.L. and D.R.M.; writing – review & editing, all authors; visualization, O.L., R.L., S.H., and D.R.M.; supervision, P.J.R. and D.R.M.; project administration, P.J.R. and D.R.M.; funding acquisition, H.C., P.J.R., and D.R.M.

DECLARATION OF INTERESTS

P.J.R. is a scientific co-founder of ReOx Ltd., a company developing inhibitors of the HIF hydroxylase enzymes.

INCLUSION AND DIVERSITY

We support inclusive, diverse, and equitable conduct of research.

Received: April 11, 2022
Revised: August 5, 2022
Accepted: October 20, 2022
Published: November 15, 2022

REFERENCES

Anders, S., Pyl, P.T., and Huber, W. (2015). HTSeq-a Python framework to work with high-throughput sequencing data. Bioinformatics *31*, 166–169.

Betts, G.N., Eustace, A., Patiar, S., Valentine, H.R., Irlam, J., Ramachandran, A., Merve, A., Homer, J.J., Moller-Levet, C., Buffa, F.M., et al. (2013). Prospective technical validation and assessment of intra-tumour heterogeneity of a low density array hypoxia gene profile in head and neck squamous cell carcinoma. Eur. J. cancer *49*, 156–165.

Bhandari, V., Hoey, C., Liu, L.Y., Lalonde, E., Ray, J., Livingstone, J., Lesurf, R., Shiah, Y.J., Vujcic, T., Huang, X., et al. (2019). Molecular landmarks of tumor hypoxia across cancer types. Nat. Genet. *51*, 308–318.

Bhandari, V., Li, C.H., Bristow, R.G., and Boutros, P.C.; PCAWG Consortium (2020). Divergent mutational processes distinguish hypoxic and normoxic tumours. Nat. Commun. *11*, 737.

Brugarolas, J.B., Vazquez, F., Reddy, A., Sellers, W.R., and Kaelin, W.G. (2003). TSC2 regulates VEGF through mTOR-dependent and -independent pathways. Cancer Cell *4*, 147–158.

Buffa, F.M., Harris, A.L., West, C.M., and Miller, C.J. (2010). Large meta-analysis of multiple cancers reveals a common, compact and highly prognostic hypoxia metagene. Br. J. Cancer *102*, 428–435.

Cerami, E., Gao, J., Dogrusoz, U., Gross, B.E., Sumer, S.O., Aksoy, B.A., Jacobsen, A., Byrne, C.J., Heuer, M.L., Larsson, E., et al. (2012). The cBio cancer genomics portal: an open platform for exploring multidimensional cancer genomics data. Cancer Discov *2*, 401–404.

Chi, J.T., Wang, Z., Nuyten, D.S.A., Rodriguez, E.H., Schaner, M.E., Salim, A., Wang, Y., Kristensen, G.B., Helland, A., Børresen-Dale, A.L., et al. (2006). Gene expression programs in response to hypoxia: cell type specificity and prognostic significance in human cancers. PLoS Med. *3*, e47.

Choudhry, H., Schödel, J., Oikonomopoulos, S., Camps, C., Grampp, S., Harris, A.L., Ratcliffe, P.J., Ragoussis, J., and Mole, D.R. (2014). Extensive regulation of the non-coding transcriptome by hypoxia: role of HIF in releasing paused RNApol2. EMBO Rep. *15*, 70–76.

Conway, J.R., Lex, A., and Gehlenborg, N. (2017). UpSetR: an R package for the visualization of intersecting sets and their properties. Bioinformatics *33*, 2938–2940.

Dale, R.K., Pedersen, B.S., and Quinlan, A.R. (2011). Pybedtools: a flexible Python library for manipulating genomic datasets and annotations. Bioinformatics *27*, 3423–3424.

Duan, C. (2016). Hypoxia-inducible factor 3 biology: complexities and emerging themes. Am. J. Physiol. Cell Physiol. *310*, C260–C269.

Elvidge, G.P., Glenny, L., Appelhoff, R.J., Ratcliffe, P.J., Ragoussis, J., and Gleadle, J.M. (2006). Concordant regulation of gene expression by hypoxia and 2-oxoglutarate-dependent dioxygenase inhibition: the role of HIF-1alpha, HIF-2alpha, and other pathways. J. Biol. Chem. *281*, 15215–15226.

Epstein, A.C., Gleadle, J.M., McNeill, L.A., Hewitson, K.S., O'Rourke, J., Mole, D.R., Mukherji, M., Metzen, E., Wilson, M.I., Dhanda, A., et al. (2001). C. elegans EGL-9 and mammalian homologs define a family of dioxygenases that regulate HIF by prolyl hydroxylation. Cell *107*, 43–54.

Eustace, A., Mani, N., Span, P.N., Irlam, J.J., Taylor, J., Betts, G.N.J., Denley, H., Miller, C.J., Homer, J.J., Rojas, A.M., et al. (2013). A 26-gene hypoxia signature predicts benefit from hypoxia-modifying therapy in laryngeal cancer but not bladder cancer. Clin. Cancer Res. *19*, 4879–4888.

Gao, J., Aksoy, B.A., Dogrusoz, U., Dresdner, G., Gross, B., Sumer, S.O., Sun, Y., Jacobsen, A., Sinha, R., Larsson, E., et al. (2013). Integrative analysis of complex cancer genomics and clinical profiles using the cBioPortal. Sci Signal *6*, l1.

Gao, W., Li, W., Xiao, T., Liu, X.S., and Kaelin, W.G., Jr. (2017). Inactivation of the PBRM1 tumor suppressor gene amplifies the HIF-response in VHL-/- clear cell renal carcinoma. Proc. Natl. Acad. Sci. USA *114*, 1027–1032.

Ghazoui, Z., Buffa, F.M., Dunbier, A.K., Anderson, H., Dexter, T., Detre, S., Salter, J., Smith, I.E., Harris, A.L., and Dowsett, M. (2011). Close and stable relationship between proliferation and a hypoxia metagene in aromatase inhibitor-treated ER-positive breast cancer. Clin. Cancer Res. *17*, 3005–3012.

Halle, C., Andersen, E., Lando, M., Aarnes, E.K., Hasvold, G., Holden, M., Syljuåsen, R.G., Sundfør, K., Kristensen, G.B., Holm, R., et al. (2012). Hypoxia-induced gene expression in chemoradioresistant cervical cancer revealed by dynamic contrast-enhanced MRI. Cancer Res. *72*, 5285–5295.

Hanahan, D., and Weinberg, R.A. (2011). Hallmarks of cancer: the next generation. Cell *144*, 646–674.

Hao, Y., Hao, S., Andersen-Nissen, E., Mauck, W.M., 3rd, Zheng, S., Butler, A., Lee, M.J., Wilk, A.J., Darby, C., Zager, M., et al. (2021). Integrated analysis of multimodal single-cell data. Cell *184*, 3573–3587.e29.

Hewitson, K.S., McNeill, L.A., Riordan, M.V., Tian, Y.M., Bullock, A.N., Welford, R.W., Elkins, J.M., Oldham, N.J., Bhattacharya, S., Gleadle, J.M., et al. (2002). Hypoxia-inducible factor (HIF) asparagine hydroxylase is identical to factor inhibiting HIF (FIH) and is related to the cupin structural family. J. Biol. Chem. *277*, 26351–26355.

Höckel, M., and Vaupel, P. (2001). Tumor hypoxia: definitions and current clinical, biologic, and molecular aspects. J. Natl. Cancer Inst. *93*, 266–276.

Hower, V., Evans, S.N., and Pachter, L. (2011). Shape-based peak identification for ChIP-Seq. BMC Bioinformatics *12*, 15.

Hu, Z., Fan, C., Livasy, C., He, X., Oh, D.S., Ewend, M.G., Carey, L.A., Subramanian, S., West, R., Ikpatt, F., et al. (2009). A compact VEGF signature associated with distant metastases and poor outcomes. BMC Med. *7*, 9.

Hudson, C.C., Liu, M., Chiang, G.G., Otterness, D.M., Loomis, D.C., Kaper, F., Giaccia, A.J., and Abraham, R.T. (2002). Regulation of hypoxia-inducible factor 1alpha expression and function by the mammalian target of rapamycin. Mol. Cell Biol. *22*, 7004–7014.

Ivan, M., Kondo, K., Yang, H., Kim, W., Valiando, J., Ohh, M., Salic, A., Asara, J.M., Lane, W.S., and Kaelin, W.G., Jr. (2001). HIFalpha targeted for VHL-mediated destruction by proline hydroxylation: implications for O2 sensing. Science *292*, 464–468.

Jaakkola, P., Mole, D.R., Tian, Y.M., Wilson, M.I., Gielbert, J., Gaskell, S.J., von Kriegsheim, A., Hebestreit, H.F., Mukherji, M., Schofield, C.J., et al. (2001). Targeting of HIF-alpha to the von Hippel-Lindau ubiquitylation complex by O2-regulated prolyl hydroxylation. Science *292*, 468–472.

Kaelin, W.G., Jr., and Ratcliffe, P.J. (2008). Oxygen sensing by metazoans: the central role of the HIF hydroxylase pathway. Mol. Cell *30*, 393–402.

Kim, D., Paggi, J.M., Park, C., Bennett, C., and Salzberg, S.L. (2019). Graph-based genome alignment and genotyping with HISAT2 and HISAT-genotype. Nat. Biotechnol. *37*, 907–915.

Kim, N., Kim, H.K., Lee, K., Hong, Y., Cho, J.H., Choi, J.W., Lee, J.I., Suh, Y.L., Ku, B.M., Eum, H.H., et al. (2020). Single-cell RNA sequencing demonstrates the molecular and cellular reprogramming of metastatic lung adenocarcinoma. Nat. Commun. *11*, 2285.

Krishna, C., DiNatale, R.G., Kuo, F., Srivastava, R.M., Vuong, L., Chowell, D., Gupta, S., Vanderbilt, C., Purohit, T.A., Liu, M., et al. (2021). Single-cell sequencing links multiregional immune landscapes and tissue-resident T cells in ccRCC to tumor topology and therapy efficacy. Cancer Cell *39*, 662–677.e6.

Lando, D., Peet, D.J., Gorman, J.J., Whelan, D.A., Whitelaw, M.L., and Bruick, R.K. (2002). FIH-1 is an asparaginyl hydroxylase enzyme that regulates the transcriptional activity of hypoxia-inducible factor. Genes Dev. *16*, 1466–1471.

Lau, K.W., Tian, Y.M., Raval, R.R., Ratcliffe, P.J., and Pugh, C.W. (2007). Target gene selectivity of hypoxia-inducible factor-alpha in renal cancer cells is conveyed by post-DNA-binding mechanisms. Br J Cancer *96*, 1284–1292.

Li, H., Handsaker, B., Wysoker, A., Fennell, T., Ruan, J., Homer, N., Marth, G., Abecasis, G., and Durbin, R.; 1000 Genome Project Data Processing Subgroup (2009). The sequence alignment/Map format and SAMtools. Bioinformatics *25*, 2078–2079.

Louphrasitthiphol, P., Ledaki, I., Chauhan, J., Falletta, P., Siddaway, R., Buffa, F.M., Mole, D.R., Soga, T., and Goding, C.R. (2019). MITF controls the TCA cycle to modulate the melanoma hypoxia response. Pigment Cell Melanoma Res. *32*, 792–808.

Love, M.I., Huber, W., and Anders, S. (2014). Moderated estimation of fold change and dispersion for RNA-seq data with DESeq2. Genome Biol. *15*, 550.

Madissoon, E., Wilbrey-Clark, A., Miragaia, R.J., Saeb-Parsy, K., Mahbubani, K.T., Georgakopoulos, N., Harding, P., Polanski, K., Huang, N., Nowicki-Osuch, K., et al. (2019). scRNA-seq assessment of the human lung, spleen, and esophagus tissue stability after cold preservation. Genome Biol. *21*, 1.

Mahon, P.C., Hirota, K., and Semenza, G.L. (2001). FIH-1: a novel protein that interacts with HIF-1alpha and VHL to mediate repression of HIF-1 transcriptional activity. Genes Dev. *15*, 2675–2686.

Maxwell, P.H., Pugh, C.W., and Ratcliffe, P.J. (2001). Activation of the HIF pathway in cancer. Curr. Opin. Genet. Dev. *11*, 293–299.

Maxwell, P.H., Wiesener, M.S., Chang, G.W., Clifford, S.C., Vaux, E.C., Cockman, M.E., Wykoff, C.C., Pugh, C.W., Maher, E.R., and Ratcliffe, P.J. (1999). The tumour suppressor protein VHL targets hypoxia-inducible factors for oxygen-dependent proteolysis. Nature *399*, 271–275.

Mole, D.R., Blancher, C., Copley, R.R., Pollard, P.J., Gleadle, J.M., Ragoussis, J., and Ratcliffe, P.J. (2009). Genome-wide association of hypoxia-inducible factor (HIF)-1alpha and HIF-2alpha DNA binding with expression profiling of hypoxia-inducible transcripts. J. Biol. Chem. *284*, 16767–16775.

Ortiz-Barahona, A., Villar, D., Pescador, N., Amigo, J., and del Peso, L. (2010). Genome-wide identification of hypoxia-inducible factor binding sites and target genes by a probabilistic model integrating transcription-profiling data and in silico binding site prediction. Nucleic Acids Res. *38*, 2332–2345.

Ortmann, B.M., Burrows, N., Lobb, I.T., Arnaiz, E., Wit, N., Bailey, P.S.J., Jordon, L.H., Lombardi, O., Peñalver, A., McCaffrey, J., et al. (2021). The HIF complex recruits the histone methyltransferase SET1B to activate specific hypoxia-inducible genes. Nat. Genet. *53*, 1022–1035.

Ragnum, H.B., Vlatkovic, L., Lie, A.K., Axcrona, K., Julin, C.H., Frikstad, K.M., Hole, K.H., Seierstad, T., and Lyng, H. (2015). The tumour hypoxia marker pimonidazole reflects a transcriptional programme associated with aggressive prostate cancer. Br. J. Cancer *112*, 382–390.

Raudvere, U., Kolberg, L., Kuzmin, I., Arak, T., Adler, P., Peterson, H., and Vilo, J. (2019). g:Profiler: a web server for functional enrichment analysis and conversions of gene lists (2019 update). Nucleic Acids Res. *47*, W191–W198.

Schmid, V., Lafleur, V.N., Lombardi, O., Li, R., Salama, R., Colli, L., Choudhry, H., Chanock, S., Ratcliffe, P.J., and Mole, D.R. (2019). Co-incidence of RCC-susceptibility polymorphisms with HIF cis-acting sequences supports a pathway tuning model of cancer. Sci. Rep. *9*, 18768.

Schödel, J., Mole, D.R., and Ratcliffe, P.J. (2013). Pan-genomic binding of hypoxia-inducible transcription factors. Biol. Chem. *394*, 507–517.

Schodel, J., Oikonomopoulos, S., Ragoussis, J., Pugh, C.W., Ratcliffe, P.J., and Mole, D.R. (2010). High-resolution genome-wide mapping of HIF-binding sites by ChIP-seq. Blood *117*, e207–e217.

Seigneuric, R., Starmans, M.H.W., Fung, G., Krishnapuram, B., Nuyten, D.S.A., van Erk, A., Magagnin, M.G., Rouschop, K.M., Krishnan, S., Rao, R.B., et al. (2007). Impact of supervised gene signatures of early hypoxia on patient survival. Radiother. Oncol. *83*, 374–382.

Semenza, G.L. (2003). Targeting HIF-1 for cancer therapy. Nat. Rev. Cancer *3*, 721–732.

Semenza, G.L. (2010). Defining the role of hypoxia-inducible factor 1 in cancer biology and therapeutics. Oncogene *29*, 625–634.

Semenza, G.L. (2012a). Hypoxia-inducible factors in physiology and medicine. Cell *148*, 399–408.

Semenza, G.L. (2012b). Hypoxia-inducible factors: mediators of cancer progression and targets for cancer therapy. Trends Pharmacol. Sci. *33*, 207–214.

Smythies, J.A., Sun, M., Masson, N., Salama, R., Simpson, P.D., Murray, E., Neumann, V., Cockman, M.E., Choudhry, H., Ratcliffe, P.J., and Mole, D.R. (2019). Inherent DNA-binding specificities of the HIF-1alpha and HIF-2alpha transcription factors in chromatin. EMBO Rep. *20*, e46401.

Sørensen, B.S., Toustrup, K., Horsman, M.R., Overgaard, J., and Alsner, J. (2010). Identifying pH independent hypoxia induced genes in human squamous cell carcinomas in vitro. Acta Oncol. *49*, 895–905.

Subramanian, A., Tamayo, P., Mootha, V.K., Mukherjee, S., Ebert, B.L., Gillette, M.A., Paulovich, A., Pomeroy, S.L., Golub, T.R., Lander, E.S., and Mesirov, J.P. (2005). Gene set enrichment analysis: a knowledge-based approach for interpreting genome-wide expression profiles. Proc. Natl. Acad. Sci. USA *102*, 15545–15550.

The_ENCODE_Consortium (2016). ENCODE Guidelines and Best Practices for RNA-Seq. Revised December 2016.

The_Encode_Consortium (2017). ENCODE Guidelines for Experiments Generating ChIP-Seq Data January 2017.

Tiana, M., Acosta-Iborra, B., Puente-Santamaría, L., Hernansanz-Agustin, P., Worsley-Hunt, R., Masson, N., García-Rio, F., Mole, D., Ratcliffe, P., Wasserman, W.W., et al. (2018). The SIN3A histone deacetylase complex is required for a complete transcriptional response to hypoxia. Nucleic Acids Res. *46*, 120–133.

Toustrup, K., Sørensen, B.S., Nordsmark, M., Busk, M., Wiuf, C., Alsner, J., and Overgaard, J. (2011). Development of a hypoxia gene expression classifier with predictive impact for hypoxic modification of radiotherapy in head and neck cancer. Cancer Res. *71*, 5923–5931.

Treins, C., Giorgetti-Peraldi, S., Murdaca, J., Semenza, G.L., and Van Obberghen, E. (2002). Insulin stimulates hypoxia-inducible factor 1 through a phosphatidylinositol 3-kinase/target of rapamycin-dependent signaling pathway. J. Biol. Chem. *277*, 27975–27981.

Vaupel, P., and Mayer, A. (2007). Hypoxia in cancer: significance and impact on clinical outcome. Cancer Metastasis Rev. *26*, 225–239.

Vaupel, P., Mayer, A., and Höckel, M. (2004). Tumor hypoxia and malignant progression. Methods Enzymol. *381*, 335–354.

Wenger, R.H., Stiehl, D.P., and Camenisch, G. (2005). Integration of oxygen signaling at the consensus HRE. Sci. STKE *2005*, re12.

WHO_International_Agency_for_Research_on_Cancer (2020). Globocan 2020: all cancers. In CANCER FACT SHEETS.

Wiesener, M.S., Jurgensen, J.S., Rosenberger, C., Scholze, C.K., Horstrup, J.H., Warnecke, C., Mandriota, S., Bechmann, I., Frei, U.A., et al. (2003). Widespread hypoxia-inducible expression of HIF-2alpha in distinct cell populations of different organs. Faseb J *17*, 271–273.

Winter, S.C., Buffa, F.M., Silva, P., Miller, C., Valentine, H.R., Turley, H., Shah, K.A., Cox, G.J., Corbridge, R.J., Homer, J.J., et al. (2007). Relation of a hypoxia metagene derived from head and neck cancer to prognosis of multiple cancers. Cancer Res. *67*, 3441–3449.

Xia, X., and Kung, A.L. (2009). Preferential binding of HIF-1 to transcriptionally active loci determines cell-type specific response to hypoxia. Genome Biol. *10*, R113.

Xia, X., Lemieux, M.E., Li, W., Carroll, J.S., Brown, M., Liu, X.S., and Kung, A.L. (2009). Integrative analysis of HIF binding and transactivation reveals its role in maintaining histone methylation homeostasis. Proc. Natl. Acad. Sci. USA *106*, 4260–4265.

Xiao, Y., Hsiao, T.H., Suresh, U., Chen, H.I.H., Wu, X., Wolf, S.E., and Chen, Y. (2014). A novel significance score for gene selection and ranking. Bioinformatics *30*, 801–807.

Yang, L., Roberts, D., Takhar, M., Erho, N., Bibby, B.A.S., Thiruthaneeswaran, N., Bhandari, V., Cheng, W.C., Haider, S., McCorry, A.M.B., et al. (2018). Development and validation of a 28-gene hypoxia-related prognostic signature for localized prostate cancer. EBioMedicine *31*, 182–189.

Zhang, Y., Liu, T., Meyer, C.A., Eeckhoute, J., Johnson, D.S., Bernstein, B.E., Nusbaum, C., Myers, R.M., Brown, M., Li, W., and Liu, X.S. (2008). Model-based analysis of ChIP-seq (MACS). Genome Biol. *9*, R137.

STAR★METHODS

KEY RESOURCES TABLE

REAGENT or RESOURCE	SOURCE	IDENTIFIER
**Antibodies**		
Purified mouse monoclonal to HIF-1α (Western blotting)	BD Transduction Laboratories	RRID: AB_398272; Cat# 610959
Purified rabbit monoclonal to HIF-2α (Western blotting)	Cell Signaling	RRID: AB_10898028; Cat# 7096S
Purified rabbit polyclonal to HIF-1β (Western blotting)	Cell Signaling	RRID: AB_10694232; Cat# 5537S
Rabbit polyclonal antisera to HIF-1α (ChIP-seq)	Lau et al., 2007	PM14
Rabbit polyclonal antisera to HIF-2α (ChIP-seq)	Lau et al., 2007; Wiesener et al., 2003	PM9
Rabbit polyclonal antisera to HIF-1β (ChIP-seq)	Novus Biologicals	RRID: AB_10003150; Cat# NB100-110
Purified rabbit polyclonal to CA9	Abcam	RRID: AB_2066533; Cat# ab15086
Purified rabbit polyclonal to VHL	Cell Signaling	RRID: AB_2716279; Cat# 68547S
Purified mouse monoclonal to β-actin conjugated to HRP	Abcam	RRID: AB_867494; Cat# ab49900
TotalSeq-A anti-human Hashtag 1 (GTCAACTCTTTAGCG)	Biolegend	RRID: AB_2750015; Cat# 394601
TotalSeq-A anti-human Hashtag 2 (TGATGGCCTATTGGG)	Biolegend	RRID: AB_2750016; Cat# 394603
TotalSeq-A anti-human Hashtag 3 (TTCCGCCTCTCTTTG)	Biolegend	RRID: AB_2750017; Cat# 394605
TotalSeq-A anti-human Hashtag 4 (AGTAAGTTCAGCGTA)	Biolegend	RRID: AB_2750018; Cat# 394607
TotalSeq-A anti-human Hashtag 5 (AAGTATCGTTTCGCA)	Biolegend	RRID: AB_2750019; Cat# 394609
**Chemicals, peptides, and recombinant proteins**		
Formaldehyde	Sigma-Aldrich	Cat# F8775-4X25ML
RNase A	Thermo Fisher	Cat# EN0531
Proteinase K (approx 18.2mg/mL)	Sigma-Aldrich	Cat# 3115887001
Protein A agarose beads	Millipore	Cat# 16–156
cOmplete protease inhibitor cocktail	Roche	Cat# 11836145001
TrypLE Express	Thermo Fisher	Cat# 12604013
Collagenase II	Thermo Fisher	Cat# 17101015
DNase I	Sigma-Aldrich	Cat# 11284932001
Fetal bovine serum	Sigma-Aldrich	Cat# F7524-500ML
**Critical commercial assays**		
MinElute PCR purification kit	Qiagen	Cat# 28006
PrepXTM DNA Library Kit	Takara	Cat# 640102
mirVana miRNA Isolation Kit	Thermo Fisher	Cat# AM1560
RNeasy Plus Mini Kit	Qiagen	Cat# 74134
RNase-free DNase Set	Qiagen	Cat# 79254
RNase-Free Turbo DNase	Ambion	Cat# AM2238
ScriptSeq v2 RNA-seq Kit	Epicentre	Cat# SSV21124
NEBNext Ultra II Directional RNA Library Prep Kit for Illumina	NEB	Cat# E7765S/L

(*Continued on next page*)

Continued

REAGENT or RESOURCE	SOURCE	IDENTIFIER
Deposited data		
ChIP-seq analysis of HIF-1α and HIF-2α in hypoxic PC3, T47D, A549 and HCT-116 cell lines	This paper	GEO: GSE200203
RNA-seq analysis of gene expression in normoxic and hypoxic PC3, T47D, A549 and HCT-116 cell lines	This paper	GEO: GSE200204
Single cell RNA-seq analysis of gene expression in normoxic/hypoxic primary normal kidney cultures and normoxic ccRCC tumor cultures	This paper	GEO: GSE200207
ChIP-seq analysis of HIF-1β in hypoxic PC3, T47D, A549 and HCT-116 cell lines	Schmid et al., (2019)	GEO: GSE130989
ChIP-seq analysis of HIF-1α, HIF-2α and HIF-1β in hypoxic HepG2 cells and in normoxic RCC4 cells	Smythies et al., (2019)	GEO: GSE120885
ChIP-seq analysis of HIF-1α, HIF-2α and HIF-1β in hypoxic HeLa cells	Ortmann et al., (2021)	GEO: GSE159128
RNA-seq analysis of gene expression in normoxic and hypoxic HepG2 and RCC4/VHL cell lines	Smythies et al., (2019)	GEO: GSE120886
RNA-seq analysis of gene expression in normoxic and hypoxic HeLa cells	Ortmann et al., (2021)	GEO: GSE169087
RNA-seq analysis of gene expression in normoxic and hypoxic HUVEC cells	Tiana et al., (2018)	GEO: GSE89840
RNA-seq analysis of gene expression in normoxic and hypoxic mel501 cells	Louphrasitthiphol et al., (2019)	GEO: GSE95280
Single cell RNA-seq analysis of gene expression in normal and tumor cells from patients with early and late stage lung adenocarcinoma	Kim et al., (2020)	GEO: GSE131907
Single cell RNA-seq analysis of gene expression in normal and tumor cells from patients with clear cell kidney cancer	Krishna et al., (2021); https://trace.ncbi.nlm.nih.gov/Traces/index.html?view=analysis&acc=SRZ190804	NCBI: PRJNA705464
Single cell RNA-seq analysis of gene expression in spleen, esophagus and lung samples subject to different durations of cold ischaemia	Madissoon et al., (2019); https://www.tissuestabilitycellatlas.org	NCBI: PRJEB31843
Pan cancer RNA-seq analysis of gene expression in tumor and patient-matched normal tissue from the TCGA database	http://cancergenome.nih.gov/	N/A
Pan cancer RNA-seq, mutation and CNV analysis of gene expression in tumor tissue from the TCGA database	https://www.cbioportal.org; Cerami et al., 2012; Gao et al., 2013	N/A
Experimental models: Cell lines		
A549	ECACC; Sigma-Aldrich	RRID: CVCL_0023; Cat# 86012804-1VL
HCT116	ECACC; Sigma-Aldrich	RRID: CVCL_0291; Cat# 91091005-1VL
T47D	ECACC; Sigma-Aldrich	RRID: CVCL_0553; Cat# 85102201-1VL
HepG2	ATCC	RRID: CVCL_0027; Cat# HB-8065
PC3	Validated by STR genotyping	RRID: CVCL_0035
RCC4	Gift from C.H. Buys; validated by detection of the VHL gene mutation (chr3:10,183,841 G > del) in RNA-seq data	RRID: CVCL_0498
RCC4/VHL	Maxwell et al., (1999)	RRID: CVCL_2706

(*Continued on next page*)

Continued

REAGENT or RESOURCE	SOURCE	IDENTIFIER
Normal kidney cells	Schmid et al., (2019); Oxford Radcliffe Biobank (ORB)	ORB ID: 110326; Lab ID: N3
ccRCC tumor cells - culture 1	Schmid et al., (2019); Oxford Radcliffe Biobank (ORB)	ORB ID: 110324; Lab ID: T3A
ccRCC tumor cells - culture 2	Schmid et al., (2019); Oxford Radcliffe Biobank (ORB)	ORB ID: 110325; Lab ID: T3B
Software and algorithms		
BWA (0.7.5a-r405)	https://github.com/lh3/bwa	N/A
SAMtools (0.1.19)	Li et al., (2009)	N/A
BEDTools (2.17.0)	Dale et al., (2011)	N/A
Picard tools (2.0.1)	http://broadinstitute.github.io/picard/	N/A
T-PIC (Tree shape Peak Identification for ChIP-Seq)	Hower et al., (2011)	N/A
MACS (model-based analysis of ChIP-seq)	Zhang et al., (2008)	N/A
TrimGalore (0.3.3)	https://github.com/FelixKrueger/TrimGalore	N/A
HISAT2 (2.05)	Kim et al. (2019); http://daehwankimlab.github.io/hisat2/	N/A
HTSeq (0.5.4p3)	Anders et al., (2015)	N/A
DESeq2	Love et al., (2014)	N/A
Seurat (4.0.3)	Hao et al., (2021)	N/A
InferCNV	https://github.com/broadinstitute/inferCNV	N/A
GSEA (gene set enrichment analysis)	Subramanian et al., (2005); Xiao et al., (2014)	N/A
gProfiler	Raudvere et al., (2019)	N/A
UpSetR (1.4.0)	Conway et al., (2017)	N/A
R (4.0.5)	https://www.r-project.org/foundation/	N/A

RESOURCE AVAILABILITY

Lead contact

Further information and requests for resources and reagents should be directed to and will be fulfilled by the lead contact David Mole (david.mole@ndm.ox.ac.uk).

Materials availability

This study did not generate new unique reagents.

Data and code availability

- Bulk RNA-seq data, ChIP-seq data and single-cell RNA-seq data generated in this study have been deposited at GEO and are publicly available as of the date of publication. The accession numbers are listed in the key resources table.
- This paper also analyzes existing, publicly available data. The accession numbers are listed in the key resources table.
- This paper does not report original code.
- Any additional information required to reanalyze the data reported in this paper is available from the lead contact upon request.

EXPERIMENTAL MODEL AND SUBJECT DETAILS

Human cell lines

A549, HCT116 and T47D cells were purchased from ECACC; HepG2 cells were purchased from ATCC; and PC3 cells were validated by STR genotyping. RCC4 cells were a gift from C.H. Buys. A549 and PC3 cells were grown in Ham's F12K medium; HCT116 cells were grown in McCoy's 5a medium; and HepG2, RCC4, RCC4-VHL and T47D cells were grown in DMEM. All cell lines were grown with 100 U/mL penicillin, 100 mg/mL streptomycin, 2mM L-glutamine and 10% fetal bovine serum (Sigma-Aldrich) and regularly tested for mycoplasma infection. All cells were maintained in 10cm plates at 37°C in a humidified incubator with 5% CO_2. When cells

reached approximately 80% confluency, cells were washed in PBS before 2mL of 0.05% Trypsin-EDTA (Sigma-Aldrich) was added for 3–10 min. A portion of the suspension was re-plated to maintain sub-confluency. Cells were kept in culture for approximately a month before being discarded and a new, early passage vial revived.

Primary cell cultures

Primary renal cell cultures were generated from freshly excised ccRCC tissue and tumor-adjacent normal kidney from a male subject (age unknown) undergoing radical nephrectomy as part of cancer treatment (Schmid et al., 2019). The subject gave their informed consent for inclusion before they participated in the study. The study was conducted in accordance with the Declaration of Helsinki, and the protocol was approved by the Ethics Committee of Oxford University Hospitals NHS Foundation Trust. Briefly, tissue blocks were washed in Hank's Balanced Salt Solution (HBSS) before being minced using scalpels. Tissue was digested by incubating in HBSS supplemented with 193U/mL Collagenase II and 3.33ug/mL DNase I for one hour at 37°C with vigorous shaking every 5–10 min. Suspensions were sequentially passed through 100um, 70um and 40um cell strainer to remove debris and undissociated tissue. Cell pellets were washed in growth medium (DMEM/F12 1:1, supplemented with L-glutamax, penicillin-streptomycin, insulin-transferrin-sodium selenite, 4 ng/mL triiodo-L-thyronine, 100 ng/mL epidermal growth factor, 36 ng/mL hydrocortisone and 10% fetal bovine serum) and plated on gelatinized dishes in growth medium. Cells were maintained at 37°C in a humidified incubator with 5% CO_2. When cells reached 80–100% confluency, cells were washed in PBS before being removed from dishes using 0.05% Trypsin-EDTA (Sigma-Aldrich) for 3–5 min. A portion of the suspension was re-plated to sub-confluency. Experiments were performed after the second or third passage.

METHOD DETAILS

Western blotting

Cell lysates were prepared in Urea-SDS lysis buffer (6.7M Urea, 10% glycerol, 1%SDS, 10mM Tris/HCl pH 6.8) supplemented with freshly added cOmplete protease inhibitor cocktail (Roche), phosphatase inhibitor cocktails 2 and 3 (Sigma-Aldrich), and 1mM DTT. Lysates were sonicated using a Bioruptor (Diagenode) at medium intensity for three cycles of 15s on/30s off, before being syringed 10 times through a 21G needle. Protein concentrations were determined by BCA assay (Pierce) and normalized. Samples were prepared for SDS-PAGE by adding Laemmli buffer and 0.1M DTT, then were resolved on AnyKD Mini-PROTEAN TGX precast gels (Bio-Rad) using mini-PROTEAN tetra cells (Bio-Rad). Proteins were transferred to PVDF membranes using mini-trans-blot cells (Bio-Rad). All Western blotting solutions were prepared in PBST (PBS containing 0.1% tween). Membranes were blocked in either 5% BSA fraction V or 5% milk for 1 h, before being incubated in primary antibodies for 2h at room temperature. Primary antibodies used were anti-HIF-1α (BD cat no. 610959), anti-HIF-2α (Cell Signaling cat no. 7096S), anti-HIF-1β (Cell Signaling cat. no. 5537S), anti-CA9 (Abcam cat no. ab15086), anti-VHL (Cell Signaling cat no. 68547S) and anti-β-actin (Abcam cat no. ab49900). HRP-conjugated secondary antibodies (all Dako) were incubated for an hour. Membranes were developed using SuperSignal West Dura Extended Duration Substrate (Thermo Scientific) and imaged using a Bio-Rad Chemidoc.

ChIP-seq

One or two 15cm plates of cells were used for each ChIP. The protocol was performed on ice/at 4°C unless otherwise stated. Protein-DNA complexes were cross-linked by adding 1% formaldehyde (Sigma-Aldrich) in growth medium to cells on tissue culture plates for 10–12 min with gentle agitation. The reaction was quenched by adding 125mM glycine and incubating with gentle agitation for 10–12 min at room temperature. Cells were washed twice in PBS and then removed from plates by scraping. Cells were centrifuged and resuspended in 500ul lysis buffer per plate (50mM Tris pH 8.1, 10mM EDTA, 1% SDS) supplemented with freshly added cOmplete protease inhibitor cocktail (Roche) at 2X concentration. After 10 min, samples were diluted 1:1 with ChIP dilution buffer (16.7mM Tris pH 8.1, 167mM NaCl, 1.2mM EDTA, 0.01% SDS, 1.1% Triton X-100) and then added to 15mL Bioruptor Plus TPX sonication tubes (1mL sample per tube). Samples were sonicated using a Diagenode Bioruptor Plus water bath sonicator using sonication cycles of 15 s on/15 s off for 15–30 min at high intensity. The sonication time was optimized for each cell line such that the majority of chromatin fragments lay in the range of 150–500bp, as measured on a Tapestation (Agilent) using D1000 reagents. Samples were centrifuged at 13000 rpm and the supernatant collected, before being diluted 6-fold in ChIP dilution buffer. Protein A agarose beads (Millipore) were washed twice using ChIP dilution buffer and by rotating on an end-over-end rotator for 5 min 40ul of 50% of bead slurry was added per ChIP sample then incubated on an end-over-end rotator for 1 h (to pre-clear lysates). Samples were centrifuged and the supernatant was collected. 15ul of anti-HIF-1α (PM14), 15ul of anti-HIF-2α (PM9), or 10ul of anti-HIF-1β (Novus Biologicals NB100-110) was added per ChIP, or 10ul of rabbit pre-immunization serum as a negative control. Samples were incubated overnight on an end-over-end rotator. 90ul of 50% bead slurry was added to each ChIP and incubated on an end-over-end rotator for 1.5 h. Samples were centrifuged at 380g for 8 min to collect the beads and the supernatant was discarded. The following wash steps were performed by incubating samples in each buffer on an end-over-end rotator for 5 min per wash. Samples were washed once in low salt wash buffer (20mM Tris pH 8.1, 150mM NaCl, 2mM EDTA, 0.1% SDS, 1% Triton X-100), once in high salt wash buffer (20mM Tris pH 8.1, 500mM NaCl, 2mM EDTA, 0.1% SDS, 1% Triton X-100), once in LiCl wash buffer (10mM Tris pH 8.1, 250mM LiCl, 1mM EDTA, 1% sodium deoxycholate, 1% Igepal), then twice in TE buffer (10mM Tris pH 8.0, 1mM EDTA). The TE washes were performed at room temperature. 120ul of freshly made elution buffer (0.1M $NaHCO_3$, 1%SDS) was added to each ChIP and incubated in a thermomixer

at room temperature/1400rpm for 15 min. Samples were centrifuged, the supernatant collected, and another 120ul added to perform a second elution as above, to obtain 240ul eluate per ChIP. Elution buffer was added to input samples up to 240ul and then input samples were processed in parallel to ChIP samples as below, with care being taken to avoid contaminating ChIP samples with input samples. 12.5ul of 4M NaCl was added and incubated in a thermomixer with a heated lid at 65°C/1400rpm overnight to reverse cross-links. Samples were allowed to cool to room temperature before 2.2ul of proteinase K (Sigma-Aldrich) was added and incubated in a thermomixer with a heated lid at 45°C/1400rpm for 4 h 1ul of RNaseA (Thermo Fisher) was added and incubated in a thermomixer at 37°C/1400rpm for 30 min 1mL of buffer PB (Qiagen) and 10ul of 3M sodium acetate pH 5.2 was added per sample, before being purified using the MinElute PCR purification kit (Qiagen) according to manufacturer's instructions. DNA was eluted in 20ul nuclease-free water. A small aliquot (2ul) was diluted 16-fold and used in qPCR reactions to test for enrichment of canonical HIF binding sites as part of quality control. Automated library preparation was performed using the Apollo prep system (Takara, PrepX Comp ILMN 32i DNA Lib 96. Cat number 640102.) All ChIP-seq experiments were performed in duplicate in accordance with ENCODE consortium guidelines (The_Encode_Consortium, 2017).

ChIP-seq analysis

Adapter sequences were trimmed as above. Reads were aligned to GRCh37 using BWA (0.7.5a-r405). Low-quality mapping was removed (MapQ <15) using SAMtools (0.1.19) (Li et al., 2009). Reads mapping to Duke Encode blacklist regions (http://hgwdev.cse.uc sc.edu/cgi-bin/hgFileUi?db = hg19&g = wgEncodeMapability) were excluded using BEDTools (2.17.0) (Dale et al., 2011). Duplicate reads were excluded using Picard tools (2.0.1) (http://broadinstitute.github.io/picard/). Read densities were normalized and expressed as reads per kilobase per million reads (RPKM). One million random non-overlapping regions selected from ENCODE DNase Cluster II peaks (http://hgdownload.cse.ucsc.edu/goldenPath/hg19/encodeDCC/wgEncodeRegDnaseClustered/) were used as a control. ChIP-seq peaks were identified using T-PIC (Tree shape Peak Identification for ChIP-Seq) (Hower et al., 2011) and MACS (model-based analysis of ChIP-seq) (Zhang et al., 2008) in control mode. Peaks detected by both peak callers were filtered quantitatively using the total count under the peak to include only peaks that were above the 99.99th percentile of random background regions selected from the ENCODE DNase II cluster (p value <0.0001). Only peaks from each independent replicate that overlapped by at least 1 base pair (BEDTools v2.17.0 (Dale et al., 2011)) were considered.

Bulk RNA-seq

Total RNA was prepared using the mirVana miRNA Isolation Kit (Life Technologies) or RNeasy Plus Mini kit (Qiagen) and treated with RNase-Free Turbo DNase (Ambion) or RNase-free DNase Set (Qiagen) according to manufacturers' instructions. RNA integrity was assessed using RNA Reagents and RNA Screentapes on a Tapestation as part of quality control. PolyA + RNA libraries were then prepared using the ScriptSeq v2 RNA-seq Kit (Epicentre, Madison, WI, USA) or the NEBNext Ultra II Directional RNA Library Prep Kit for Illumina. All RNA-seq experiments were performed in triplicate in accordance with ENCODE consortium guidelines (The_ENCODE_Consortium, 2016).

Bulk RNA-seq analysis

Illumina adaptor sequences were trimmed using TrimGalore (0.3.3). Reads were aligned to Genome Reference Consortium GRCh37 (hg19) using HISAT2 (2.05) (http://daehwankimlab.github.io/hisat2/) (Kim et al., 2019). Non-uniquely mapped fragments were excluded using Picard tools (2.0.1) (http://broadinstitute.github.io/picard/). Total read counts for each UCSC-defined gene were extracted using HTSeq (0.5.4p3) (Anders et al., 2015) with "intersection-strict" mode, and significantly regulated genes were identified using DESeq2 (Love et al., 2014).

Single cell RNA-seq

Primary cell cultures were detached and made into single cell suspensions using TrypLE Express (Thermo Fisher) with pipetting. Cells were labeled with hashtag antibodies to enable multiplexing, essentially as per manufacturer's instructions (Biolegend, https://www.protocols.io/view/totalseq-a-antibodies-and-cell-hashing-with-10x-si-261geo6mol47/v1). Single cell suspensions were incubated with TotalSeq-A 'hashtag' antibodies (1ug antibody with approximately 5×10^5 cells) with the following barcode sequences: anti-human 1 (GTCAACTCTTTAGCG), anti-human 2 (TGATGGCCTATTGGG), anti-human 3 (TTCCGCCTCTCTTTG) and anti-human 4 (AGTAAGTTCAGCGTA). Cell viability and concentration were assessed using Trypan Blue and a TC10 automated cell counter (Bio-Rad). 2500 cells from each sample were pooled, loaded onto a single channel of a 10x genomics chip, and single cell 3′ RNA libraries were prepared according to manufacturer's instructions (10x genomics, GEX-v3.1 chemistry, single index).

Single cell RNA-seq analysis

scRNAseq analysis was performed using Seurat version 4.0.3 (Hao et al., 2021). Single-cell somatic large-scale chromosomal copy number alterations were calculated using inferCNV (https://github.com/broadinstitute/inferCNV).

Gene set analysis

Gene set enrichment analysis (GSEA) used 10,000 permutations, weighted enrichment score and pre-ranking of genes (Subramanian et al., 2005). Both differential expression significances according to DESeq2 and fold-difference between the two conditions were used to rank genes according to the equation (Xiao et al., 2014).

$$\pi_i = \varphi_i(-log_{10}pv_i)$$

where ui is the log2 fold-change, and Pvi is the p-value for gene i. Functional enrichment analysis of biological categories enriched in gene lists was performed using the gProfiler web server (Raudvere et al., 2019). Gene set overlap was examined using UpSetR version 1.4.0 (Conway et al., 2017).

TCGA RNA-seq data

FPKM-UQ normalized RNA-seq data for 9,760 primary tumor samples and 730 normal samples were obtained from https://portal.gdc.cancer.gov/repository on 07.09.2021 using the gdc-client version 1.5.0 and the following advanced filters: cases.project.program.name in ["TCGA"], files.analysis.workflow_type in ["HTSeq - FPKM-UQ"], files.data_category in ["transcriptome profiling"], files.experimental_strategy in ["RNA-Seq"] and either cases.samples.sample_type in ["primary tumor"] or cases.samples.sample_type in ["solid tissue normal"].

Sequencing

Sequencing was performed on the HiSeq 2500, HiSeq 4000 or NovaSeq 6000 platforms according to Illumina protocols (Illumina).

QUANTIFICATION AND STATISTICAL ANALYSIS

All statistical analyses were performed using R version 4.0.5. All statistical details for experiments can be found in the figure legends and the results section.

Cell Reports, Volume *41*

Supplemental information

Pan-cancer analysis of tissue and single-cell HIF-pathway activation using a conserved gene signature

Olivia Lombardi, Ran Li, Silvia Halim, Hani Choudhry, Peter J. Ratcliffe, and David R. Mole

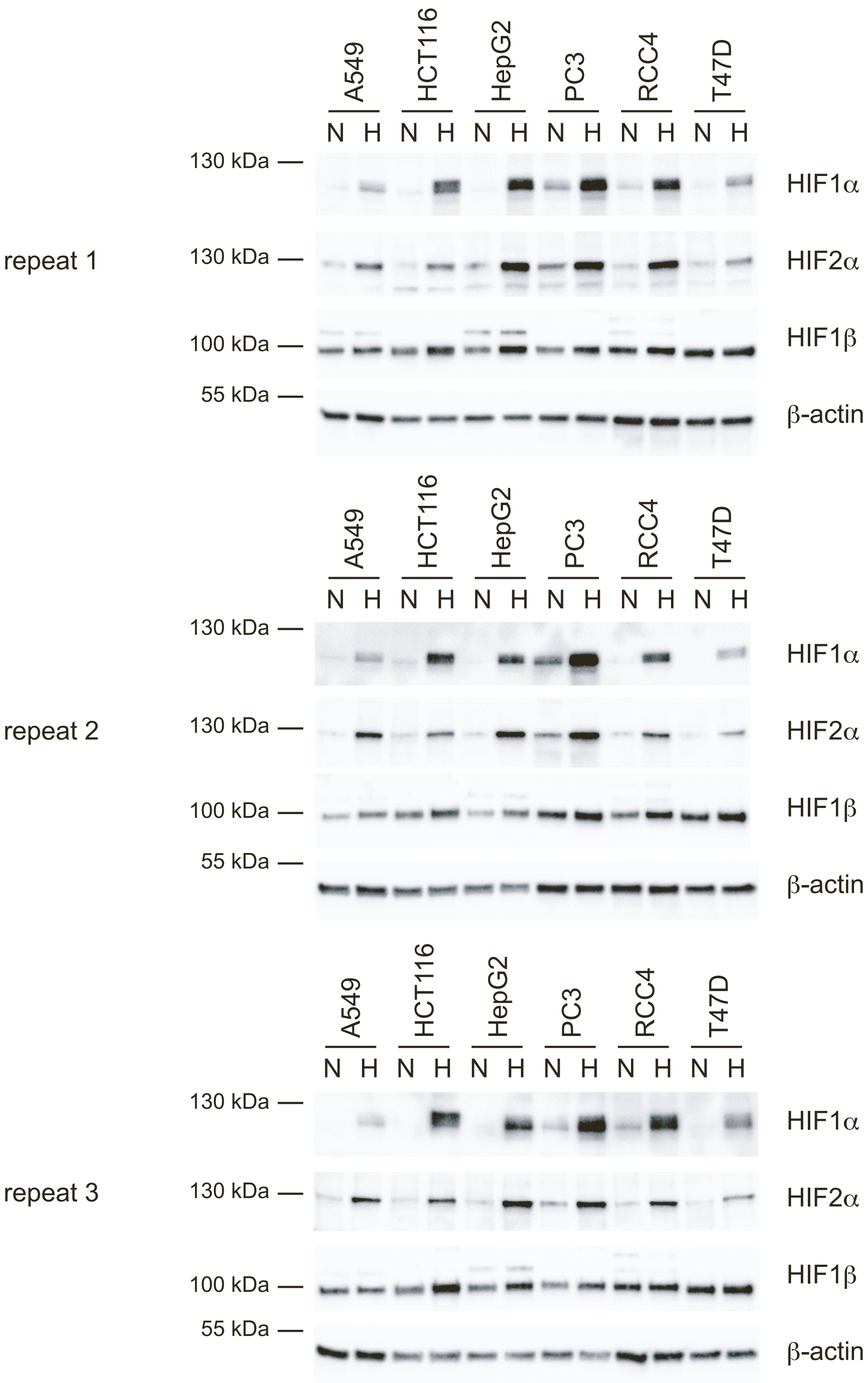

Supplemental Figure 1. HIF subunit levels in cell lines used. Related to Figure 1. Immunoblot showing relative HIF-1α, HIF-2α and HIF-1β protein levels in A549, HCT116, HepG2, PC3, RCC4 and T47D cells.

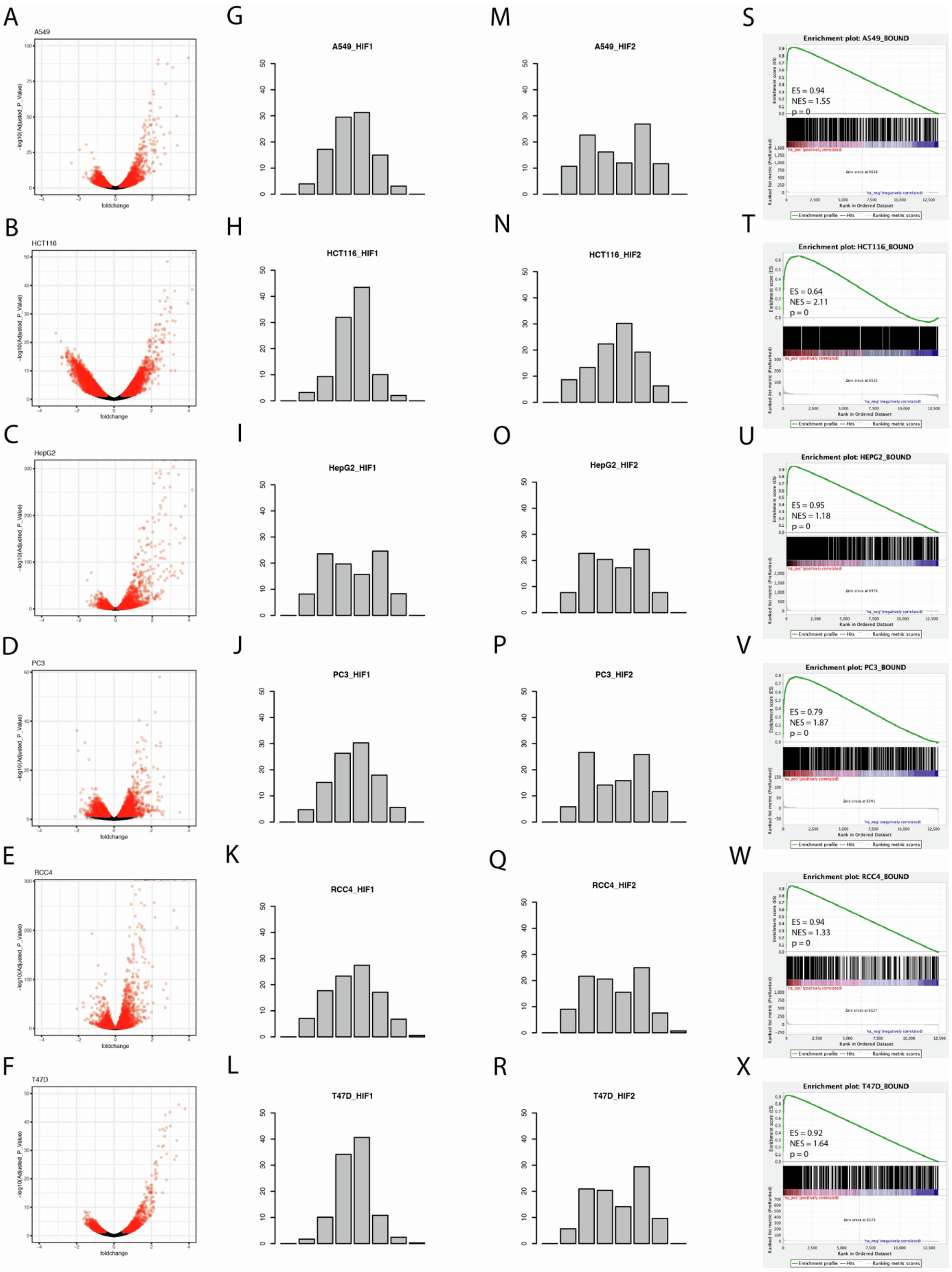

Supplemental Figure 2. Summary of RNA-seq and ChIP-seq analyses in cell lines. Related to Figure 1. Volcano plots showing log2(fold-change) and -log10(p-value) for gene expression in hypoxia and normoxia in RNA-seq analysis of (A) A549, (B) HCT116, (C) HepG2, (D) PC3, (E) RCC4+VHL and (F) T47D cells. Histograms showing frequency distribution of distance to nearest TSS for (G-L) canonical HIF-1 binding sites and (M-R) canonical HIF-2 binding sites. (S-

X) Gene set enrichment analysis (GSEA) showing the enrichment of HIF-bound genes amongst genes induced, but not suppressed by hypoxia.

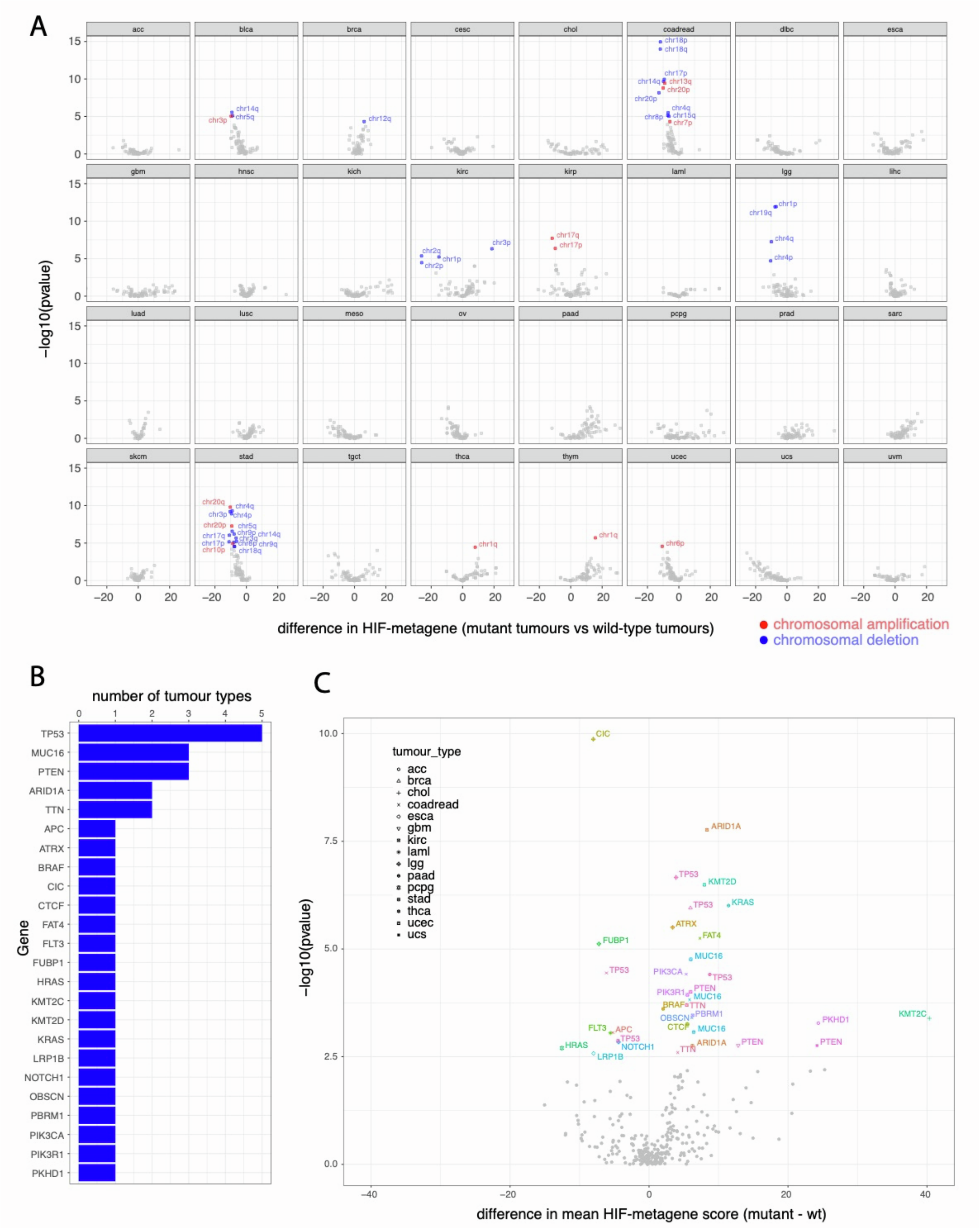

Supplemental Figure 3. Association between genetic mutation and HIF-metagene in tumors from the TCGA database. Related to Figures 3 and 4**.** (A) Volcano plots showing the association between chromosomal arm-level copy number alterations and HIF-metagene score. Red dots show the effect of chromosomal amplification, blue dots show the effect of chromosomal deletion and grey dots show non-significant associations. (B) The number of tumor types in which commonly mutated genes are associated with significantly altered HIF-metagene. (C) Volcano plot showing association between gene mutation and HIF-metagene score for the 10 most commonly mutated genes in each tumor type.

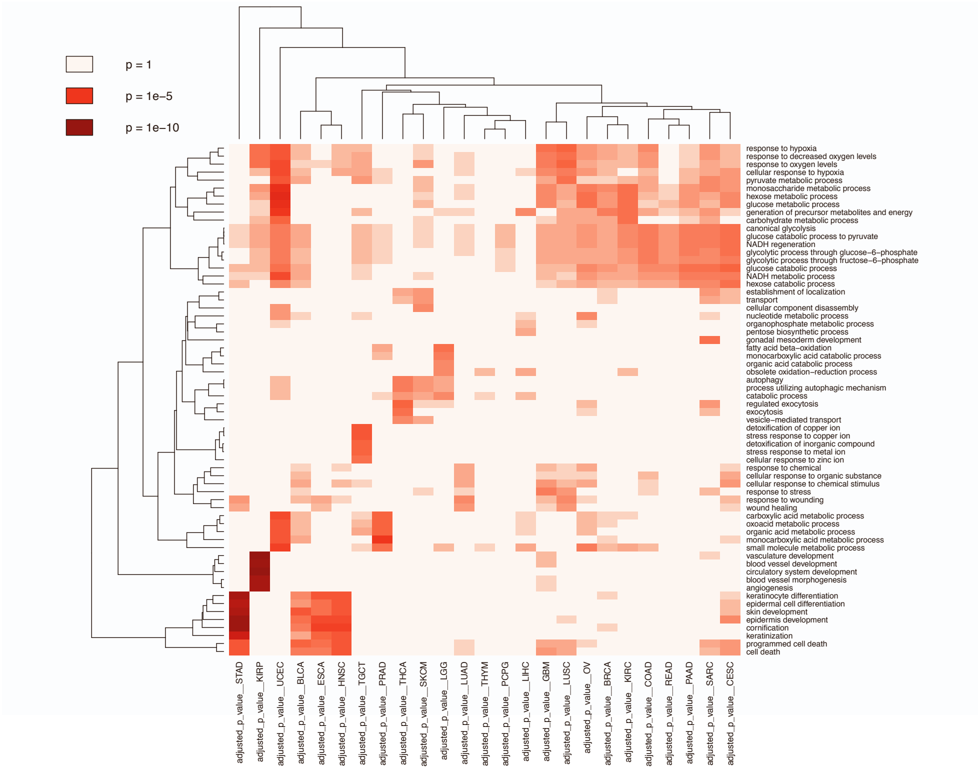

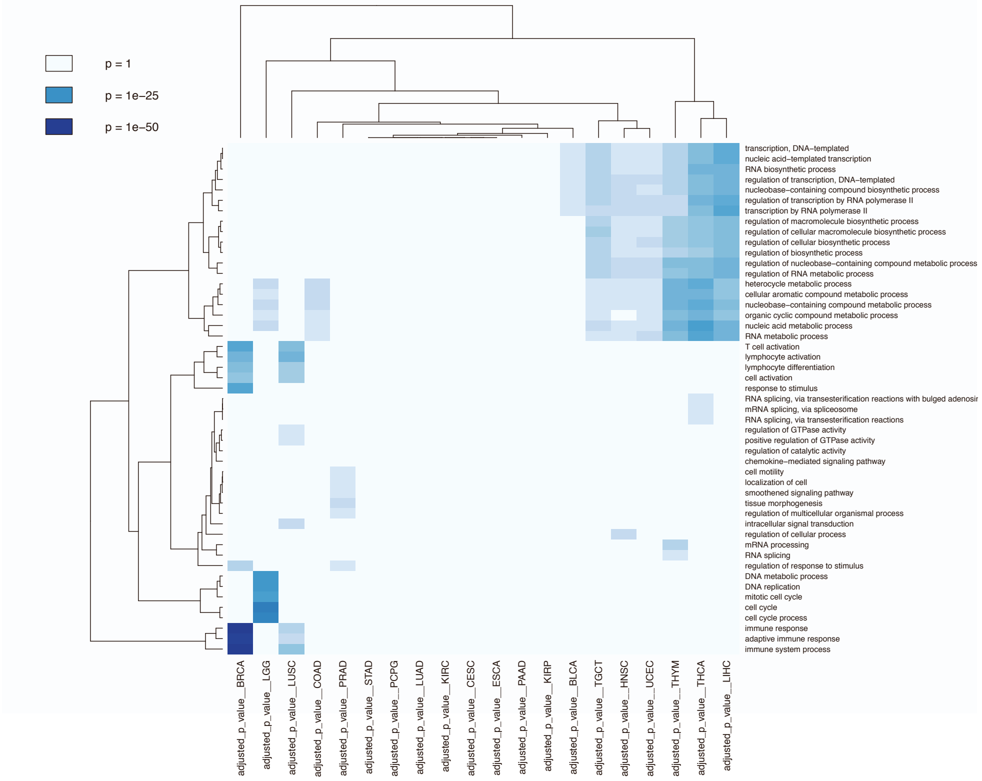

Supplemental Figure 4. Pathway enrichment for genes correlating with HIF-metagene in tumors from the TCGA database. Related to Figures 3 and 4. Heatmap showing -log10(p-value) for pathways enriched amongst (A) genes that positively correlate with the HIF-metagene in each tumor type using RNA-seq analyses from the TCGA for 9,760 tumors drawn from 32 cancer categories and (B) genes that negatively correlate with the HIF-metagene.

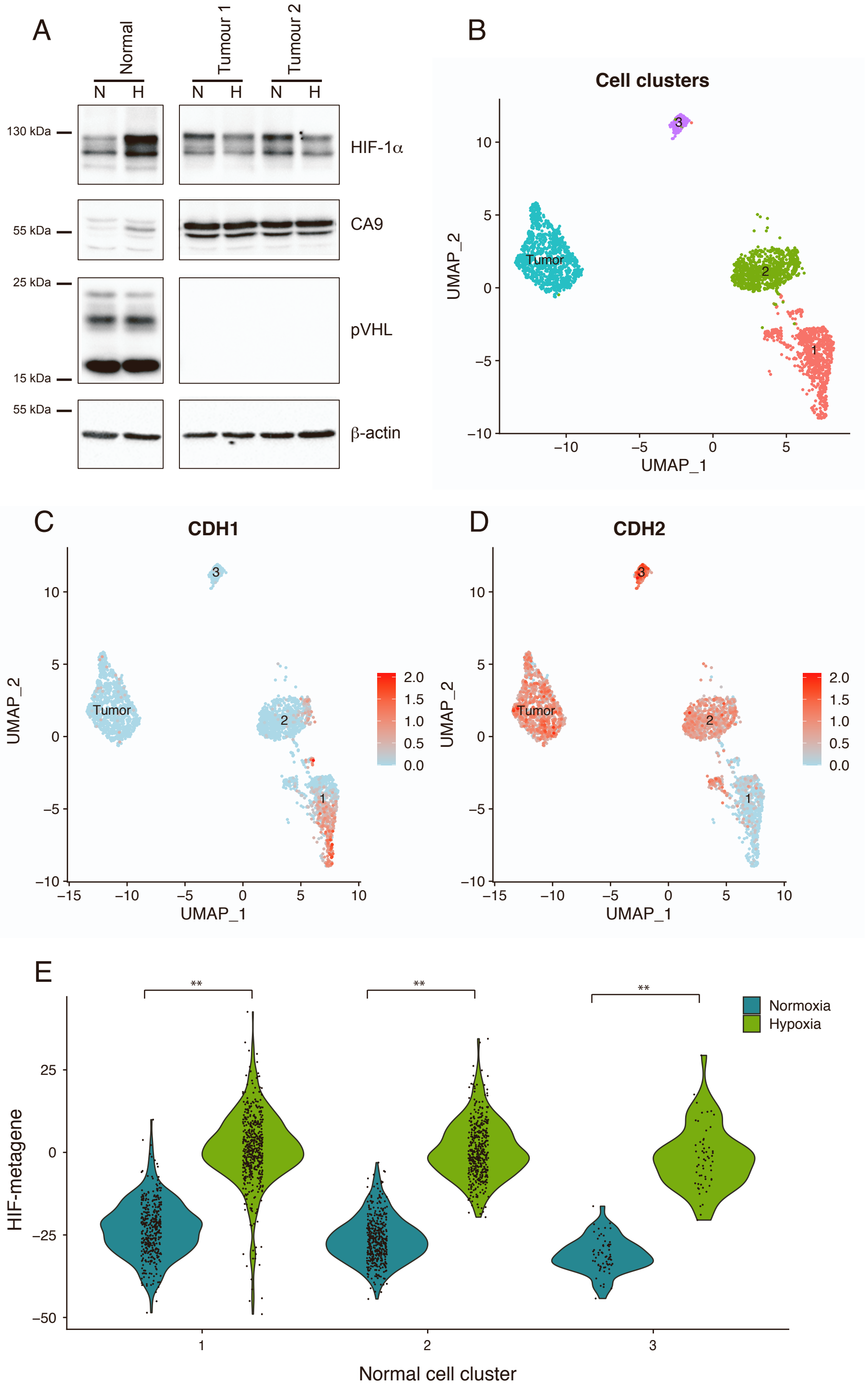

Supplemental Figure 5. Subgroup analysis of individual clusters in scRNA-seq analysis of cultured normal cells. Related to Figure 5. (A) Immunoblot showing HIF-1α, CA9, pVHL and β-actin protein levels in normoxia and hypoxia. UMAP plots showing (A) sample of origin, (B) individual clusters denoted 1=distal tubular epithelial cells, 2=proximal tubular epithelial cells, 3=non-epithelial cells and tumor=ccRCC cancer cells, (C) expression of the distal tubular marker, CDH1, and (D) expression of the proximal tubular marker, CDH2. (E) Violin plot showing HIF-metagene expression in cells from each normal cell cluster incubated in normoxia and 0.5% hypoxia for 16 hours (**, $p<10^{-16}$, Wilcoxon rank sum).

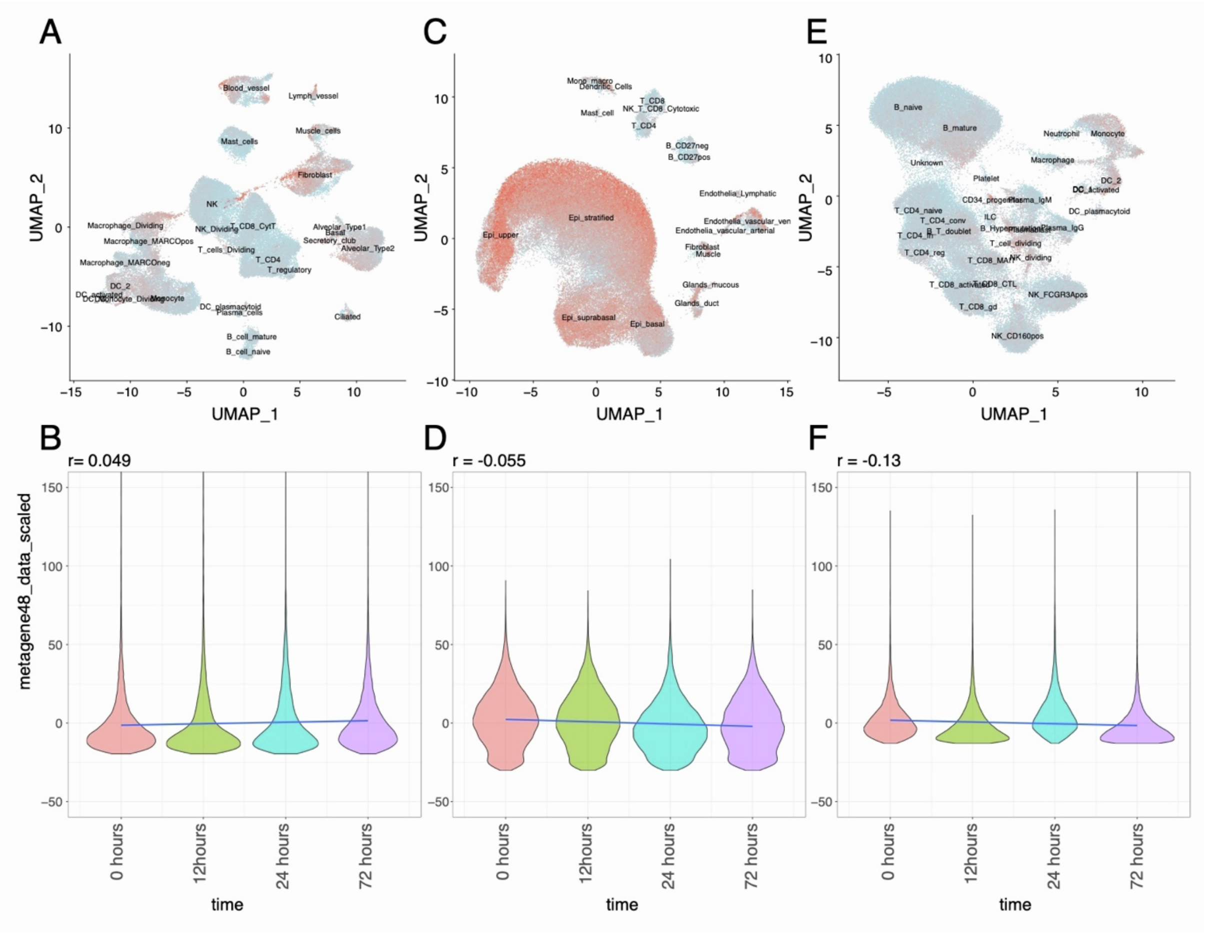

Supplemental Figure 6. **The effect of cold ischemic storage on HIF-metagene in scRNA-seq**. Related to Figures 5 and 6. (A) UMAP plot and (B) violin plot showing HIF-metagene scores in scRNAseq analysis of cells from lung samples subjected to 0, 12, 24 and 72 hours cold ischemic storage prior to processing (Madissoon et al). The same analyses in cells from (C and D) esophagus samples and (E and F) spleen samples.

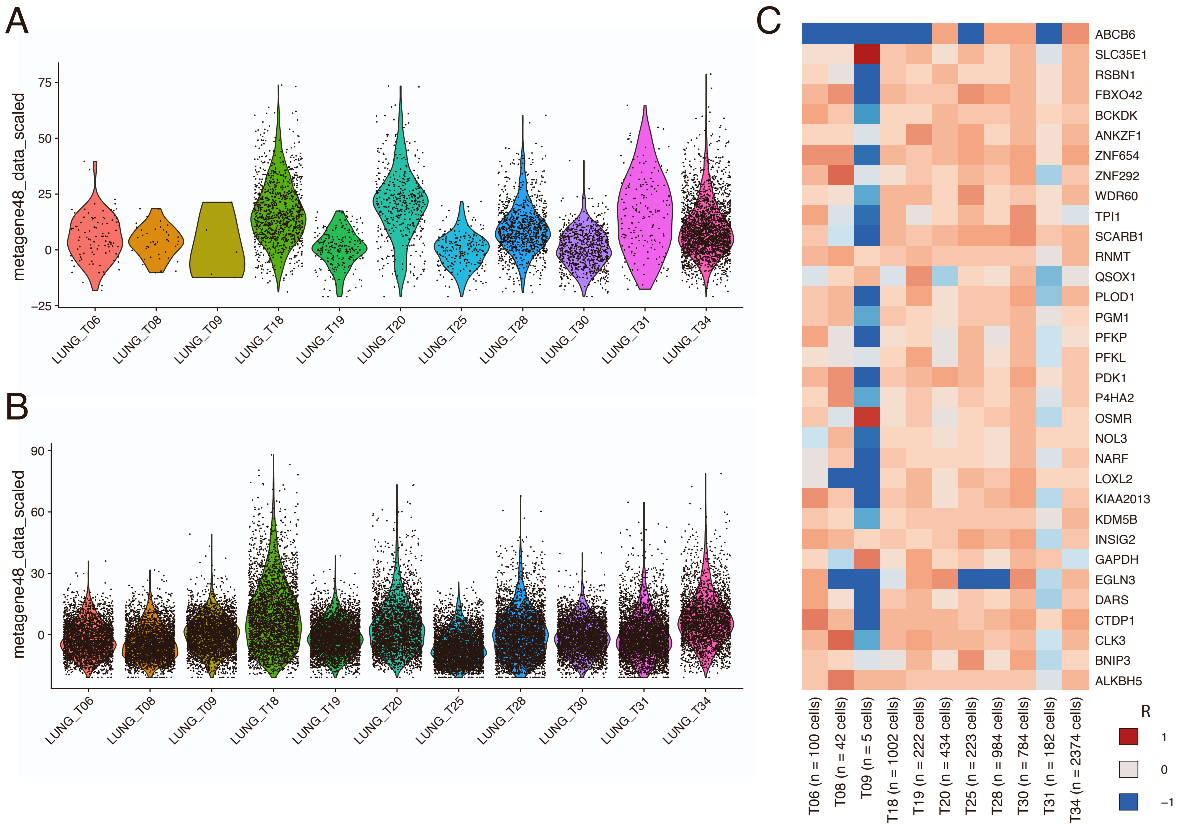

Supplemental Figure 7. HIF-metagene in individual early-stage lung cancer samples. (A) Violin plot showing HIF-metagene expression in tumor cells from each early-stage lung cancer sample in Kim et al. (B) Violin plot showing HIF-metagene expression in non-tumor cells from the same samples. (C) Heatmap showing Pearson correlation coefficient between the HIF-metagene and second-tier HIF-target genes (identified in 5/6 cancer cell lines) in tumor cells from each, individual early-stage lung cancer sample.

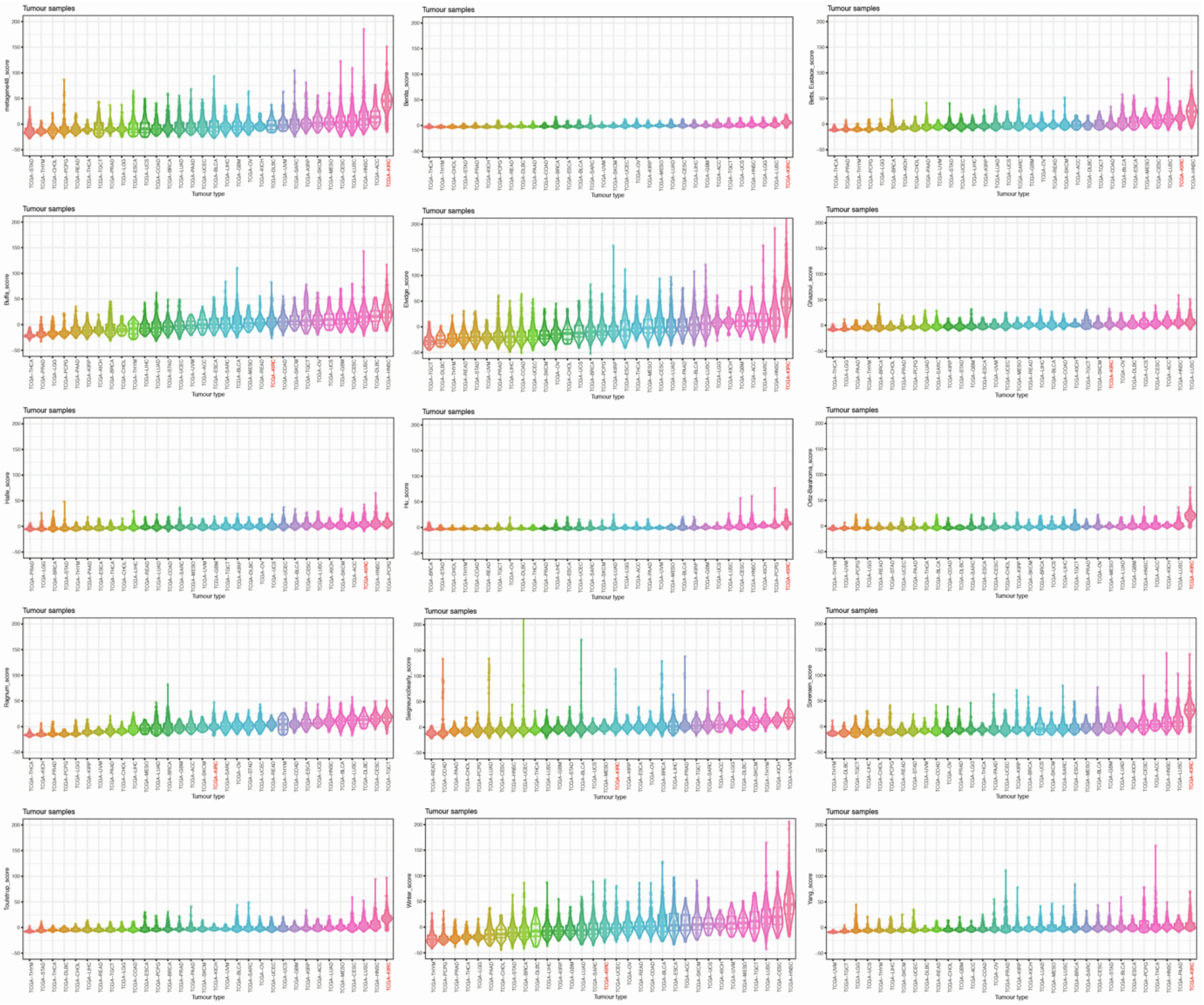

Supplemental Figure 8. Hypoxic gene expression signatures in tumors from the TCGA database. Scatter/box-and-whisker/violin plots showing metagene expression in RNA-seq analysis of 9,760 tumors from the TCGA database using genes from (A) our 48-gene HIF-metagene (B) Benita, (C) Betts and Eustace, (D) Buffa, (E) Elvidge, (F) Ghazoui, (G) Halle, (H) Hu, (I) Ortiz-Barahoma, (J) Ragnum, (K) Seineuric, (L) Sorensen, (M) Toustrup, (N) Winter and (O) Yang. Tumors are grouped according to tumor type and ranked according to median expression for that tumor type.
